# Sequence-defined donor-acceptor-donor oligo(*para*-phenylene ethynylene)s with emission across the visible spectrum

**DOI:** 10.1038/s41598-026-59109-2

**Published:** 2026-06-30

**Authors:** Qianyu Cai, Lars Boller, Michael A. R. Meier

**Affiliations:** 1 Institute of Organic Chemistry (IOC), Karlsruhe Institute of Technology (KIT), 76131 Karlsruhe, Germany; 2 Institute of Biological and Chemical Systems – Functional Molecular Systems (IBCS-FMS), Karlsruhe Institute of Technology (KIT), 76131 Karlsruhe, Germany

**Keywords:** Chemistry, Materials science, Optics and photonics, Physics

## Abstract

Donor-acceptor engineering is a widely employed strategy for π-conjugated molecules to modulate their electronic structure and photophysical behavior. Oligo(*para*-phenylene ethynylene)s (OPEs) provide a rigid and modular π-conjugated scaffold that is particularly well suited for the systematic incorporation of donor and acceptor units. In this work, a symmetric donor-acceptor-donor (D-A-D) sequence was adopted to tune the electronic structure of the OPE molecular rods. By varying the acceptor unit, two series of OPE emitters, **OPE-*x*** (3-mers) and **OPE-*x*’** (5-mers), were synthesized via Sonogashira cross-coupling reactions. With increasing acceptor strength, the emission wavelength could be tuned from the blue to the red spectral region for both applied conjugation lengths, while preserving high photoluminescence quantum yields (PLQY). By extending the degree of conjugation by a defined number of repeating units, a decrease was observed for both the Stokes shift and the emission full width at half maximum (FWHM) across the series. Further analysis aided by quantum-chemical calculations provides a molecular-level understanding of the observed photophysical trends.

**Supplementary Information:**

The online version contains supplementary material available at 10.1038/s41598-026-59109-2.

## Introduction

π-Conjugated macromolecular materials exhibit optoelectronic properties that arise from electronic delocalization along their conjugated segments. These properties are highly sensitive to molecular structure and overall architecture^1–4^. In conventional polymeric systems, statistic monomer sequence distributions and inherent dispersity result in averaged properties, limiting the extraction of accurate structure-property relationships^5^. Therefore, monodisperse sequence-defined macromolecular systems, which provide the necessary structural precision, are essential for understanding how changes at the level of individual π-conjugated units govern optoelectronic properties.

Among various conjugated macromolecules, oligo(*para*-phenylene ethynylene)s (OPEs) provide a well-suited platform for sequence-definition studies^6^. The C ≡ C triple bonds restrict rotational freedom along the backbone, leading to an extended and rigid π-conjugated scaffold. Accordingly, such structures exhibit efficient charge and energy transport^7,8^ and often display high PLQY in solution^9–11^, which has motivated their investigations in areas such as field-effect transistors^12–14^, light-emitting diodes^15–18^, photocatalysis^19^, and sensing applications.^20,21^ Moreover, the ethynylene linkages act as spacers that enable versatile substitutions of the aromatic units^22^, enabling precise sequence-control through modular synthesis. Similar to other sequence-defined macromolecules^23–25^, sequence definition in uniform OPEs is primarily achieved through iterative synthetic strategies involving stepwise monomer addition and deprotection steps^22,26^. As dictated from its chemical nature, the construction of OPE chains typically relies on Sonogashira-type cross-coupling reactions^22,26^. Such reactions are well-known for broad functional-group tolerance^27–29^ and therefore allow the incorporation of a wide variety of substituents. As a result, molecularly defined OPEs serve as promising model systems for investigating structure-property relationships in π-conjugated architectures. In addition, their pronounced molecular rigidity renders them suitable benchmark systems for fundamental experimental and theoretical investigations.

Our previous work on sequence-defined OPEs focused primarily on oligomer length variation, monomer positioning, and side-chain variation^22,26,30,31^. The present study shifts the emphasis towards electronic structure engineering through the use of donor (D) and acceptor (A) building units. To minimize the synthetic complexity, a symmetric D-A-D architecture was adopted in the first place.

**Fig. 1:**
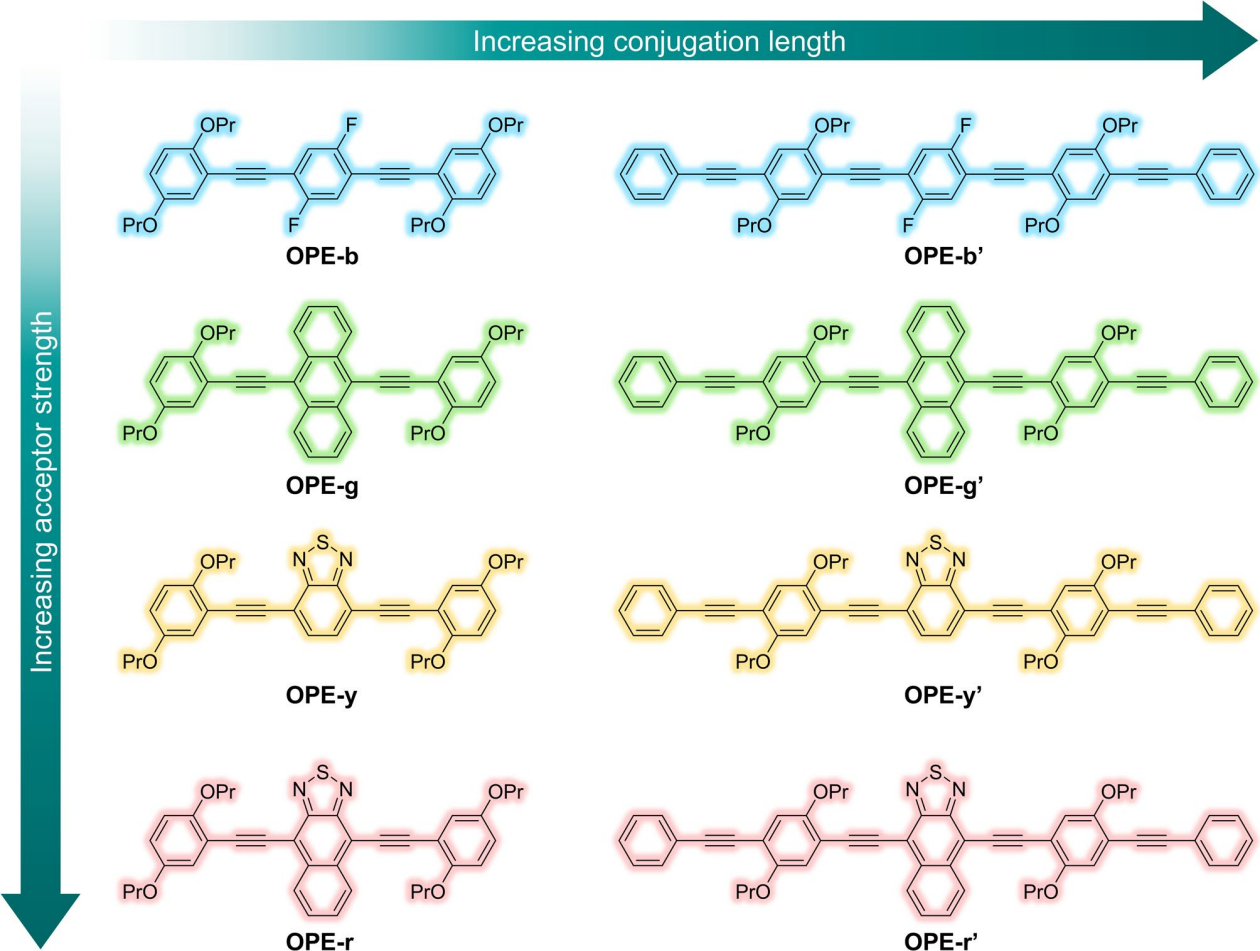
Structures of investigated OPEs. Left: the **OPE-*x*** series. Right: the **OPE-*x*’** series with extended conjugation length.

Accordingly, eight OPE emitters were developed and synthesized (Fig. 1), applying four different central acceptor units and two donor units with different conjugation length. Thus, for each central acceptor unit, two derivatives were synthesized: a classical D-A-D architecture (**OPE-*x***) and an extended analogue bearing two additional terminal phenyl groups flanking the donor units (**OPE-*x*’**). This molecular design aims to demonstrate that donor-acceptor sequences in OPE molecules can access emission across different regions of the visible spectrum. The chain length was extended by one repeating unit on both ends to investigate the impact of further structural variations on electronic properties. Quantum-chemical calculations were employed to support property investigations. Thus, the steady-state photophysical properties in solution were compared both within and between the two conjugation-length series.

## Results and discussion

### Synthetic access

**Fig. 2:**
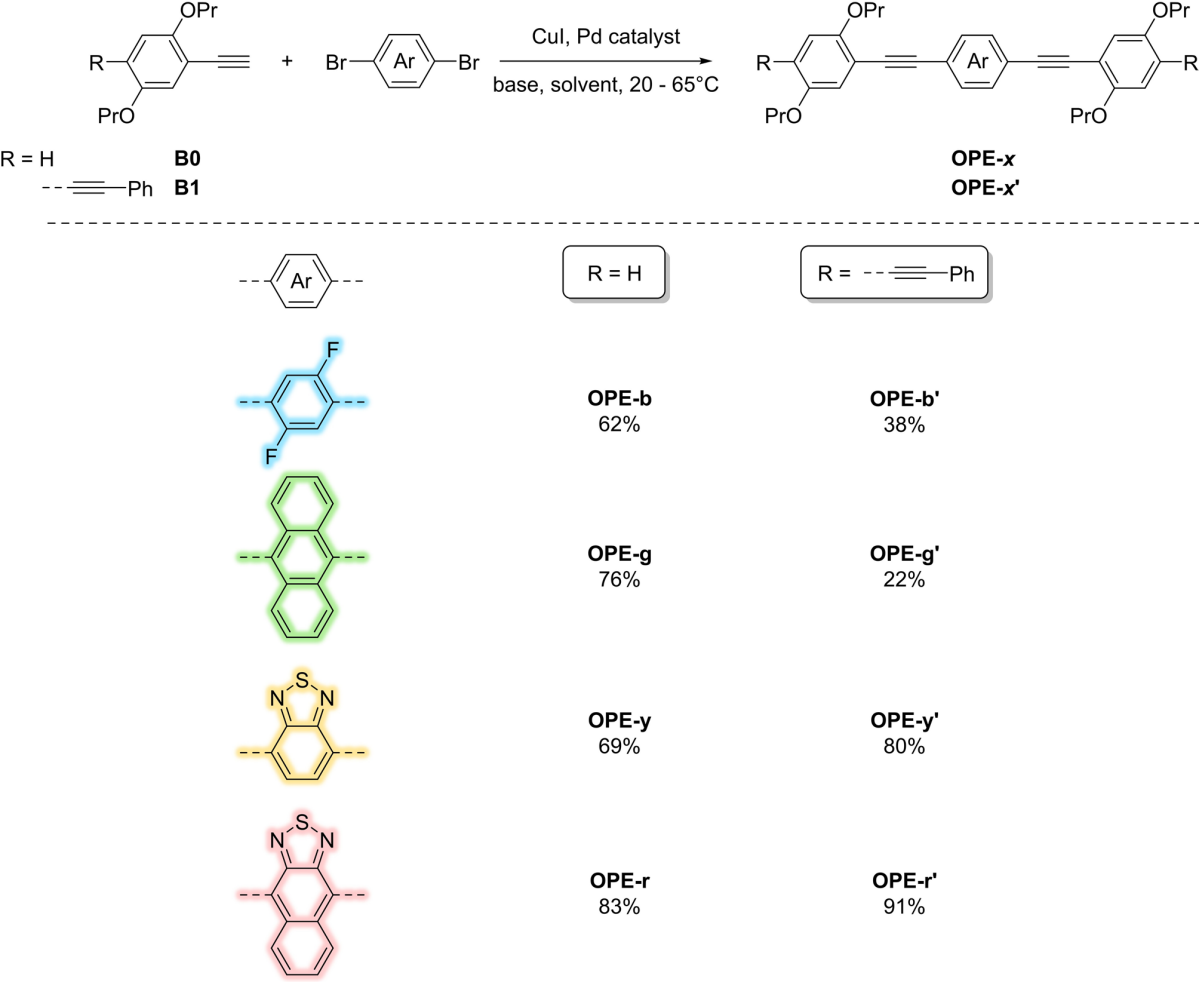
Top: general reaction for the synthesis of D-A-D-type **OPE-*x*** and **OPE-*x*’** molecules. Bottom: chemical structure of the incorporated acceptor units and isolated yield of **OPE-*x*** and **OPE-*x*’** derivatives.

The **OPE-*x*** and **OPE-*x*’** series of emitters were synthesized starting from donor building units **B0** and **B1**, respectively, which were prepared based on the earlier reports^7^. Each OPE series comprises a library of molecules incorporating different acceptor cores, including *para*-difluorobenzene, anthracene, benzothiadiazole (BTD), and naphthothiadiazole (NTD). The general reaction scheme and the isolated yields for each compound are summarized in Fig. 2, synthetic and analytical details are provided in the Supplementary Information.

**Fig. 3:**
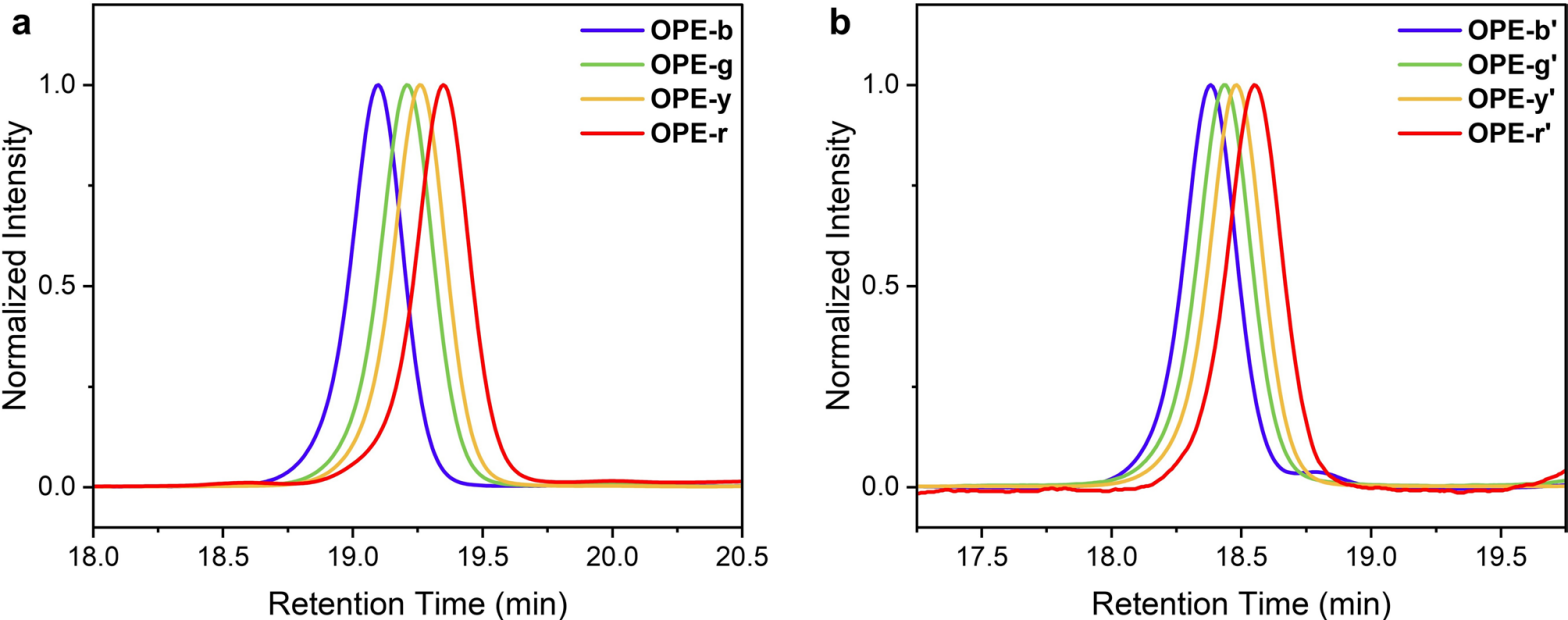
SEC traces of the purified products of **(a)** the **OPE-*x*** series and **(b)** the **OPE-*x*’** series.

The synthesis was performed on a preparative scale using established Sonogashira cross-coupling conditions^22^. Purification via Celite^®^ filtration and flash column chromatography yielded **OPE-b** as a white cotton-like solid in a yield of 62%. The structure was confirmed by nuclear magnetic resonance (NMR) spectroscopy (provided in the Supplementary Information), and its purity was verified by size-exclusion chromatography (SEC, Fig. 3a, blue trace)^32^. In contrast, the same purification procedure proved insufficient for its π-extended derivative **OPE-b’**. SEC of chromatographically purified **OPE-b’** showed a small peak (13% integration) at higher retention time next to the main product peak, indicating a species of possibly lower molecular weight (Supplementary Figure S1a, black trace). Electrospray ionization mass spectrometry (ESI-MS) suggests that this species of similar polarity and solubility as a Glaser-coupling product (**GB1**, *m*/*z* = 634.31), likely formed due to traces of contamination with oxygen (Supplementary Figure S1b). Due to difference in ionization efficiency, the higher signal intensity of **GB1** compared to the target compound **OPE-b’** in the mass spectrum does not reflect its actual abundance. As confirmed by ^1^H NMR spectroscopy of the chromatographically purified substance (Figure S1c), **OPE-b’** remained the predominant species in the mixture after careful validation. However, an additional purification step was necessary. Thus, sonification in *n-*hexane was conducted to selectively solubilize the byproduct. Indeed, the red trace in the SEC diagram (Figure S1a) showed that this treatment reduced the impurity to less than 3% of the peak area. **OPE-b’** was thereby isolated as a neon yellow powder in a moderate yield of 38% in an SEC-purity of 97%. This approach of sonification of the material in *n*-hexane was thus employed consistently as the final purification step to remove the Glaser-coupling side-products.

For all the other derivatives, precipitation from the reaction mixture was observed during stirring. After complete conversion, as indicated by thin-layer chromatography (TLC), the crude solids were collected by filtration over Celite^®^, washed with hexane(s), and taken up in dichloromethane. ^1^H NMR spectroscopy confirmed the precipitates as the target molecules, and their purity was further confirmed by SEC (Fig. 3). **OPE-g**, **OPE-g’**, **OPE-y**, **OPE-y’** were obtained as orange powders in yields ranging from 20% to 80%, while **OPE-r** and **OPE-r’** were isolated as dark purple powders in 83% and 91% yield, respectively. All compounds were fully characterized by NMR spectroscopy, infrared spectroscopy, and high-resolution mass spectrometry (HRMS). With the exception of **OPE-b’**, as discussed above, all compounds exhibited SEC purities above 99%.

### Theoretical investigations

To assess the electronic impact of the selected acceptor units and to anticipate the photophysical behavior of the resulting D-A-D systems, quantum-chemical calculations were performed using the Gaussian 16 package^33^. First, density functional theory (DFT) calculations were performed at the PBE0^34^/6-31G**^35,36^ level of theory, including Grimme’s D3 dispersion correction with Becke-Johnson damping^37,38^, to optimize the ground-state geometries, followed by wavefunction analysis^39^ to provide insight into spatial distribution and energy levels of the frontier molecular orbitals (FMOs). All calculations were carried out in gas phase. To reduce computational cost, the *n*-propyl chains were substituted with methyl groups, as their electronic effect on the conjugated core was expected to be minimal. Optimized geometries and FMO density distributions for **OPE-*x*** and **OPE-*x*’** are provided in Supplementary **Figure S16**. Within both series, the calculated LUMO energy decreases in the order *para*-difluorobenzene < anthracene < BTD < NTD, consistent with the increasingly electron-deficient character of the central unit. Between the two series, **OPE-*x*’** molecules exhibit smaller HOMO-LUMO gaps than their **OPE-*x*** analogues and thus a general red-shift in the optical transitions is expected.

**Fig. 4:**
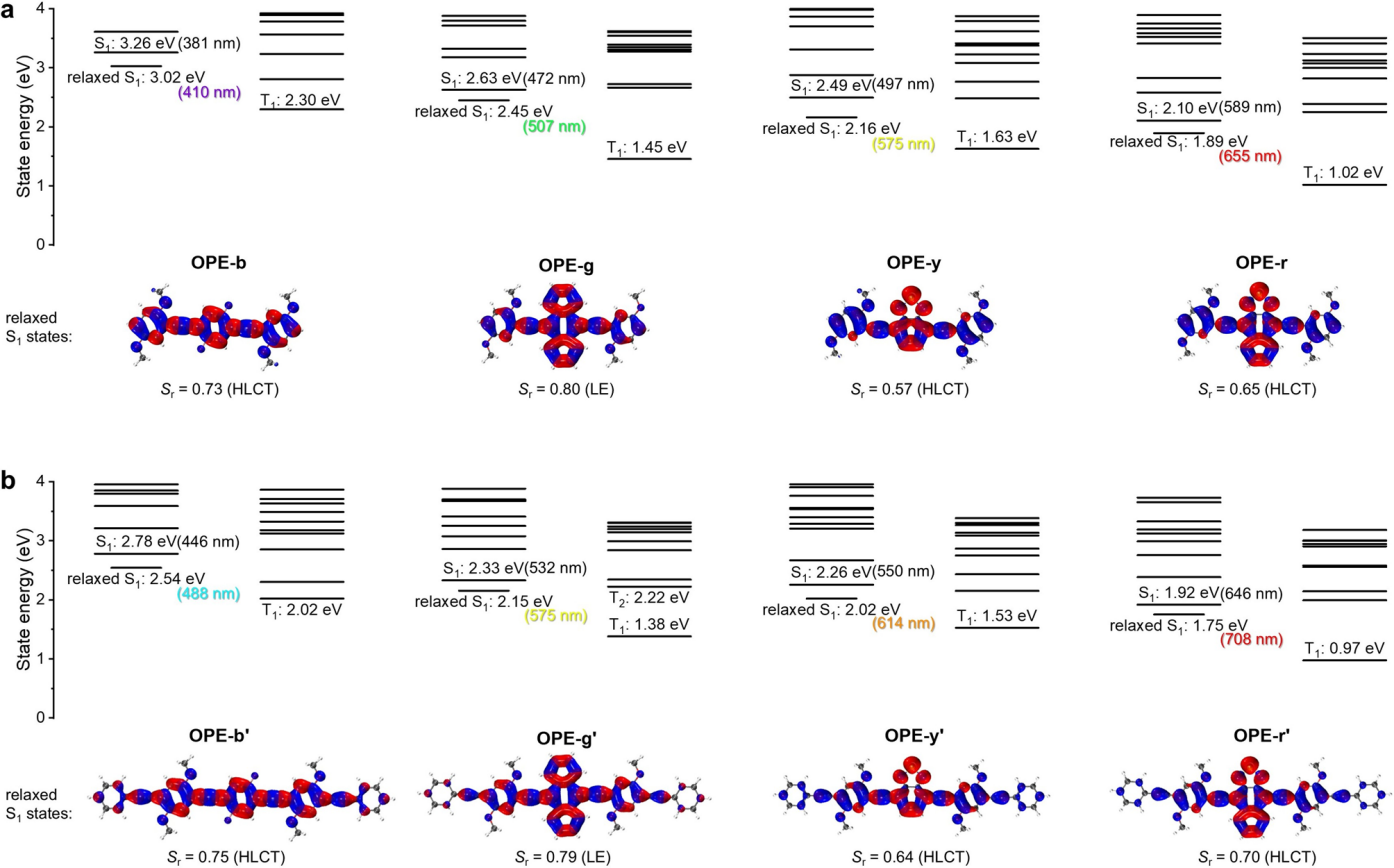
Vertical excitation energies of representative excited states and the energy of relaxed S_1_ geometry for **(a) OPE-*x*** and **(b) OPE-*x*’** series, calculated at the D3-PBE0/6-31G** level of theory. For the S_1_ levels, the corresponding wavelengths are given in parentheses. The spatial distributions of the hole (blue) and electron (red) in the relaxed S_1_ states are shown as natural transition orbitals (NTOs), along with the corresponding S_r_ index for each molecule. The orbital pictures are plotted with an isovalue of 0.02.

To gain further insight into the optical properties, time-dependent DFT (TD-DFT) calculations were performed at the corresponding level of theory (D3-PEB0/6-31G**). Vertical excitation energies were computed for the lowest 10 singlet and 10 triplet excited states of each molecule (Fig. 4, complemented by Supplementary Table S1). For each lowest singlet excited state (S_1_), a geometry optimization was carried out, followed by wavefunction analysis. The hole-electron character of the S_1_→S_0_ transition was visualized in the form of natural transition orbitals (NTOs)^39^. To quantitatively assess the extent of hole-electron separation, the orbital overlap index *S*_r_ was employed. It is defined as

1 $${S}_{\mathrm{r}}=\int\:\sqrt{{\rho\:}^{\mathrm{hole}}\left(\boldsymbol{r}\right){\rho\:}^{\mathrm{ele}}\left(\boldsymbol{r}\right)}d\boldsymbol{r}$$

where *ρ*^hole^(**r**) and *ρ*^ele^(**r**) denote the spatial distributions of hole and electron densities, respectively^40^. Based on the value of *S*_r_, the nature of the excited states is conventionally classified as charge transfer (CT) for 0-0.4, hybridized local excitation and charge transfer (HLCT) for 0.4–0.75, and local excitation (LE) for 0.75–1^41–44^.

With increasing acceptor strength, both the vertical excitation energies and the relaxed levels of S_1_ states decrease across the series from **OPE-b** to **OPE-r**. The first vertical excitation energies range from 3.26 eV to 2.10 eV, while the relaxed S_1_ levels lie between 3.02 eV and 1.89 eV. The latter corresponds to the emission wavelengths, spanning from 410 nm to 655 nm, thus covering the visible light spectrum from deep blue to red. Although **OPE-g** exhibits a similar HOMO-LUMO gap to **OPE-y** (Supplementary Figure S16a), its S_1_ level, especially after relaxation, lies significantly higher at 2.45 eV (507 nm) compared to 2.16 eV (575 nm) for **OPE-y** (Fig. 4a). When translated into wavelength (Table 1), this corresponds to a difference of approximately 70 nm, shifting the emission from the green to the yellow region. All molecules in the series show *S*_r_ values above 0.5, indicating a considerable LE character. Especially **OPE-g**, containing an anthracene core, exhibits an *S*_r_ index of 0.80 for the relaxed S_1_ geometry, which corresponds to a pure LE state according to the classification mentioned above. In contrast, **OPE-y** and **OPE-r**, two molecules with heterocyclic acceptors (BTD and NTD), show lower *S*_r_ values of 0.57 and 0.65, respectively. Their increased CT character is attributed to the polarizability and electron-withdrawing nature of the heterocycles.

As depicted in Fig. 4b, TD-DFT calculations revealed similar trends for the **OPE-*x*’** series. Due to the increased degree of π-conjugation, the energies of excited states are generally lower than their **OPE-*x*** analogues. Therefore, red-shifted vertical excitation and emission are predicted. The excited-state characteristics remain largely unaffected, as only a minor portion of the electron density is localized on the additional terminal phenyl rings.

### Steady-state optical characterization

**Fig. 5:**
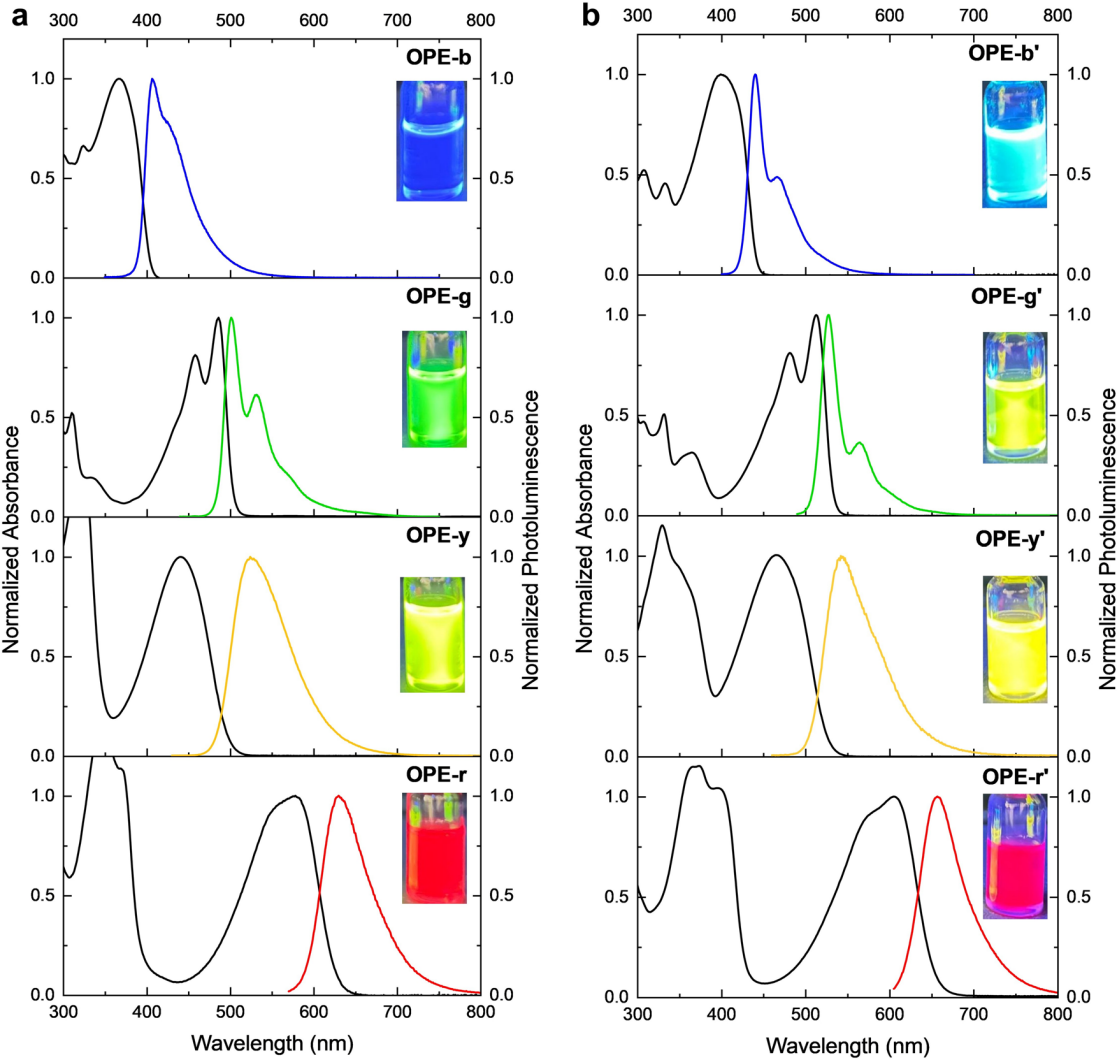
Normalized absorption and PL spectra of (**a**) the **OPE-*x*** series and (**b**) the **OPE-*x*’** series, measured in ~ 10 µM toluene solutions. From top to bottom: **(a) OPE-b**, **OPE-g**, **OPE-y**, and **OPE-r**; **(b) OPE-b’**, **OPE-g’**, **OPE-y’**, and **OPE-r’**. Absorption spectra (black lines) were normalized with respect to the lowest-energy absorption band to enable direct comparison with the corresponding PL spectra (colored lines). The inset shows photographs of the corresponding toluene solutions taken under 365 nm UV irradiation.

UV-Vis absorption, PL, and (absolute) quantum yield (Φ_PL_) measurements were carried out in dilute toluene solution (10 µM). As a non-polar solvent that mimics the environment of typical host materials in organic light-emitting diode (OLED) devices^44–46^, toluene provided sufficient solubility for the **OPE-*x*** and **OPE-*x*’** series at the required concentration. Figure 5 shows the normalized absorption and PL spectra, together with photographs of the corresponding toluene solutions taken under UV irradiation. The key spectral features and Φ_PL_ values are summarized in Table 1.

**Table 1:** Summary of steady-state photophysical properties and TD-DFT calculations for **OPE-*x*** and **OPE-*x*’** derivatives. The experiments were conducted in ~ 10 µM toluene solutions. (a) Absolute PLQY measured in non-degassed toluene solution. (b) Oscillator strength (dimensionless) given in parentheses.

Entry	λ_abs, exp_ / nm	λ_PL, exp_ (FWHM) / nm	CIE [x, y]	Δṽ_exp_ / cm^− 1^	Φ_PL_^a^	λ_S0−S1,calc_ / nm (f)^b^	λ_S1−S0,calc_ / nm (f)^b^	Δṽ_calc_ / cm^− 1^
**OPE-b**	368	407 (51)	0.157, 0.051	2600	93%	381 (1.85)	410 (2.09)	1860
**OPE-g**	486	501 (48)	0.240, 0.627	616	83%	472 (1.42)	507 (1.52)	1460
**OPE-y**	441	524 (77)	0.325, 0.614	3590	100%	497 (0.91)	575 (0.73)	2730
**OPE-r**	578	630 (66)	0.682, 0.318	1430	65%	589 (0.90)	655 (0.81)	1710
**OPE-b’**	399	440 (23)	0.150, 0.084	2340	97%	446 (3.67)	488 (4.11)	1930
**OPE-g’**	512	527 (24)	0.315, 0.656	556	83%	532 (2.96)	576 (3.28)	1440
**OPE-y’**	464	542 (71)	0.400, 0.583	3100	79%	550 (2.11)	614 (1.96)	1900
**OPE-r’**	605	658 (60)	0.715, 0.285	1330	68%	646 (1.90)	708 (1.82)	1360

**OPE-b** absorbs at 368 nm and emits blue light at 407 nm, while the π-extended analogue **OPE-b’** shows red-shifted absorption at 399 nm and blue emission at 440 nm, translating into Commission International de L’Éclairage (CIE) coordinates of (0.157, 0.051) and (0.150, 0.084), respectively (Figure S12). **OPE-g** and **OPE-g’** both absorb and emit both in green region with absorption maxima at 486 and 512 nm and PL maxima at 501 and 527 nm, respectively. Thus, **OPE-g** (616 cm^− 1^) and **OPE-g’** (556 cm^− 1^) exhibited the smallest Stokes shifts among all derivatives, accompanied by mirror-like symmetric vibronic progressions. This indicates minimal structural reorganization between the ground and excited states, consistent with the fused aromatic character of the anthracene central unit. As illustrated in Figure S17a for **OPE-g**, the overlay of the optimized S_0_ geometry (blue) and relaxed S_1_ geometry (red) reveals a high degree of structural consistency. The stronger acceptor-containing derivatives **OPE-y** and **OPE-y’** show absorption maxima at 441 nm and 464 nm, respectively, while their emission is red-shifted to 524 nm and 542 nm. Thus, **OPE-y** shows the largest Stokes shift (2730 cm^− 1^) within the series. Upon excitation, its peripheral donor units exhibit a pronounced bending towards the heterocyclic acceptor, whereas the ground-state structure shows a more linear OPE-backbone (Figure S17b). This substantial structural deviation also explains the observation that the emission maximum of **OPE-y** was red-shifted by 23 nm relative to **OPE-g**, whereas its absorption maximum was blue-shifted by 45 nm. Finally, **OPE-r** absorbs in the yellow region and emits red light at 630 nm, while **OPE-r’** absorbs in the orange region and emits a deep red light at 658 nm, translating into CIE coordinates of (0.682, 0.318) and (0.715, 0.285).

In general, the **OPE-*x*’** series showed a red-shift of approximately 20–30 nm in both absorption and PL maxima compared to **OPE-*x***, respectively. This observation is consistent with the slightly reduced HOMO-LUMO gaps attributed to the extended π-conjugation. In addition, the increased conjugation led to narrower PL spectra, as indicated by the reduced FWHM values. Notably, the FWHM of **OPE-b** (51 nm) decreased to 23 nm for **OPE-b’**. This can be associated with a weaker vibronic coupling, as displayed by the reduced shoulder peak in the emission profile^44, 47^. The PLQY measurements were conducted in non-degassed toluene solutions. The obtained Φ_PL_ values ranged from 65% to 100%, indicating overall high emission efficiencies for both series. For **OPE-y**, repeated measurements confirmed a PLQY close to unity within experimental uncertainty (< 7%)^48^. In contrast to the spectral changes, the Φ_PL_ values did not reveal a clear dependence on conjugation length within the range investigated in this work.

## Conclusion

In summary, two series of D-A-D type OPE emitters, **OPE-*x*** and **OPE-*x*’**, covering a broad range of emission colors, were successfully synthesized and characterized. Their steady-state photophysical properties were investigated in toluene and analyzed with the aid of DFT/TD-DFT calculations. Compared to their **OPE-*x*** counterparts, the elongated conjugation in the **OPE-*x*’** derivatives resulted in narrower emission bands and smaller Stokes shift, reflecting the increased rigidity and enhanced delocalization of the conjugated backbone. Notably, the green emitter **OPE-g’**, incorporating an anthracene moiety for further extended conjugation, displayed the narrowest emission spectrum (FWHM = 24 nm) and the smallest Stokes shift (556 cm^− 1^) in toluene among the investigated molecules. All emitters showed high absolute quantum yields exceeding 65%. In particular, the blue emitters (**OPE-b**: 93%, **OPE-b’**: 97%) and green emitters (**OPE-g**: 83%, **OPE-g’**: 83%) exhibited excellent quantum yields in both series. In terms of chromaticity, **OPE-b** and **OPE-b’** well matched the sRGB blue coordinate^49^, while **OPE-r’** aligned closely with the red primary suggested by BT.2020^50^.

## Supplementary Information

Below is the link to the electronic supplementary material.

Supplementary Material 1

## Data Availability

All data generated or analyzed during this study are included in this published article and its Supplementary Information files.

## References

[1] Müllen, K. & Scherf, U. Conjugated Polymers: Where we come from, where we stand, and where we Might go. *Macromol. Chem. Phys.* **224**, 2200337 (2023).

[2] Khasbaatar, A. et al. From solution to thin film: molecular assembly of π-conjugated systems and impact on (opto)electronic properties. *Chem. Rev.* **123**, 8395–8487 (2023). 37273196 10.1021/acs.chemrev.2c00905

[3] Devadiga, D., Yan, J. & Devadiga, D. Recent advances in probing electron delocalization in conjugated molecules by attached infrared reporter groups for energy conversion and storage. *ACS Appl. Energy Mater.* **8**, 1942–1963 (2025). 40018390 10.1021/acsaem.4c03246 PMC11863185

[4] Zhao, N., Jeon, S. J. & Li, Y. Cross-conjugated polymer semiconductors. *Macromol. Rapid Commun.* **46**, e00281 (2025). 40605093 10.1002/marc.202500281 PMC12447699

[5] Meier, M. A. R. & Barner-Kowollik, C. A new class of materials: sequence-defined macromolecules and their emerging applications. *Adv. Mater.* **31**, 1806027 (2019). 10.1002/adma.201806027 30600565

[6] Solleder, S. C., Schneider, R. V., Wetzel, K. S., Boukis, A. C. & Meier, M. A. R. Recent progress in the design of monodisperse, sequence-defined macromolecules. *Macromol. Rapid Commun.* **38**, 1600711 (2017). 10.1002/marc.201600711 28297122

[7] Linton, K. E., Fox, M. A., Pålsson, L. O. & Bryce, M. R. Oligo(p-phenyleneethynylene) (OPE) molecular wires: synthesis and length dependence of photoinduced charge transfer in OPEs with triarylamine and diaryloxadiazole end groups. *Chem. Eur. J.* **21**, 3997–4007 (2015). 25630530 10.1002/chem.201406080

[8] Liu, S., Peng, J., Bao, P., Shi, Q. & Lan, Z. Ultrafast Excited-State Energy Transfer in Phenylene Ethynylene Dendrimer: Quantum Dynamics with the Tensor Network Method. *J. Phys. Chem. A*. **128**, 6337–6350 (2024). 39047261 10.1021/acs.jpca.4c00322

[9] Weder, C. & Wrighton, M. S. Efficient Solid-State Photoluminescence in New Poly(2,5-dialkoxy-p-phenyleneethynylene)s. *Macromolecules* **29**, 5157–5165 (1996).

[10] Sharber, S. A. & Thomas, I. I. I. Small Changes With Big Consequences: Swapping Two Atoms In Side Chains Changes Phenylene-Ethynylene Packing And Fluorescence. *Chem. – Eur. J.* **24**, 16987–16991 (2018). 30281848 10.1002/chem.201804648

[11] Barboza-Ramos, I., Gobeze, H. B., Wherritt, D. & Schanze, K. S. Water-Soluble Poly(phenylene ethynylene)s That Contain Phosphonium Pendant Groups. *Macromolecules* **57**, 7575–7585 (2024).

[12] Kim, K. H. et al. Open-Bandgap Graphene-Based Field-Effect Transistor Using Oligo(phenylene-ethynylene) Interfacial Chemistry. *Angew. Chem.* **134**, e202209726 (2022). 10.1002/anie.202209726 PMC9826410 35969510

[13] Wang, X. et al. Electrostatic Fermi level tuning in large-scale self-assembled monolayers of oligo(phenylene–ethynylene) derivatives. *Nanoscale Horizons*. **7**, 1201–1209 (2022). 35913108 10.1039/d2nh00241h

[14] Tirgar Fakheri, M., Tehrani, M. A. & Navi, K. A novel two-input NOR logic gate using a dual-gate field effect transistor based on an OPE molecule. *J. Comput. Electron.* **24**, 51 (2025).

[15] Ervithayasuporn, V. et al. Synthesis, characterization, and OLED application of oligo(*p*-phenylene ethynylene)s with polyhedral oligomeric silsesquioxanes (POSS) as pendant groups. *Tetrahedron* **66**, 9348–9355 (2010).

[16] Usta, H. et al. Highly Efficient Deep-Blue Electroluminescence Based on a Solution-Processable A – π–D – π–A Oligo(p-phenyleneethynylene) Small Molecule. *ACS Appl. Mater. Interfaces*. **11**, 44474–44486 (2019). 31609580 10.1021/acsami.9b12971

[17] Usta, H. et al. A hybridized local and charge transfer excited state for solution-processed non-doped green electroluminescence based on oligo(p-phenyleneethynylene). *J. Mater. Chem. C*. **8**, 8047–8060 (2020).

[18] Kuttiyullathil, S., Sudhakaran, S. V., Arputharaj, D. S., Hussien, M. & Thomas, R. Tuning of Optoelectronic Properties in Oligo(phenyleneethynylene)-Based Cocrystals through Modulation of Charge-Transfer Interactions. *ACS Omega*. **10**, 52308–52319 (2025). 41244484 10.1021/acsomega.5c03330 PMC12612889

[19] Weyl, B. et al. Visible Light Excitation of Poly-(para-Phenylene Ethynylene) Enables Heterogeneous Photocatalytic Oxidations of Amines in Flow. *Angew. Chem.* **137**, e202419169 (2025). 10.1002/anie.202419169 39436200

[20] Hill, E. H., Zhang, Y., Evans, D. G. & Whitten, D. G. Enzyme-Specific Sensors via Aggregation of Charged p-Phenylene Ethynylenes. *ACS Appl. Mater. Interfaces*. **7**, 5550–5560 (2015). 25697234 10.1021/acsami.5b00185

[21] Kaafarani, D. & Karam, P. Poly(Phenylene Ethynylene) Based Thermochromic Nanoparticles with Large Fluorescence Shifts for Thermal Sensing. *ACS Appl. Nano Mater.*10.1021/acsanm.5c03252 (2025).

[22] Schneider, R. V. et al. Sequence-definition in stiff conjugated oligomers. *Sci. Rep.* **8**, 17483 (2018). 30504924 10.1038/s41598-018-35933-z PMC6269511

[23] Xu, C. et al. Regio- and sequence-controlled conjugated topological oligomers and polymers via boronate-tag assisted solution-phase strategy. *Nat. Commun.* **12**, 5853 (2021). 34615871 10.1038/s41467-021-26186-y PMC8494804

[24] Xu, H., Ye, S., Zhao, R. & Seferos, D. S. Homogeneous Synthesis of Monodisperse Sequence-Defined Conjugated Oligomers by Temperature Cycling. *Angew. Chem. Int. Ed.* **61**, e202210340 (2022). 10.1002/anie.202210340 35930340

[25] Milis, W. et al. Versatile Strategy to Develop Sequence-Defined Conjugated Macromolecules: A Powerful Tool toward Tunable Optoelectronic Properties. *ACS Macro Lett.* **13**, 1293–1303 (2024). 39284131 10.1021/acsmacrolett.4c00526

[26] Hahn, D., Schneider, R. V., Foitzik, E. & Meier, M. A. R. A Practical and Efficient Synthesis of Uniform Conjugated Rod-Like Oligomers. *Macromol. Rapid Commun.* **42**, 2000735 (2021). 10.1002/marc.202000735 33646627

[27] Mohajer, F., Heravi, M., Zadsirjan, M., Poormohammad, N. & V. & Copper-free Sonogashira cross-coupling reactions: an overview. *RSC Adv.* **11**, 6885–6925 (2021). 35423221 10.1039/d0ra10575a PMC8695108

[28] Vadakkethil Arundhathi, K., Vaishnavi, P., Aneeja, T. & Anilkumar, G. Copper-catalyzed Sonogashira reactions: advances and perspectives since 2014. *RSC Adv.* **13**, 4823–4834 (2023). 36760276 10.1039/d2ra07685c PMC9903355

[29] Yan, F., Zhang, X., Li, D., Zhu, N. & Bao, H. Recent Applications of the Sonogashira Reaction in the Synthesis of Drugs and Their Derivatives: A Review. *Appl. Organomet. Chem.* **39**, e7932 (2025).

[30] Wegelin, S. & Meier, M. A. R. Solution Self-Assembly of Branched Macromolecules Obtained via Iterative OPE Synthesis and the Passerini Three-Component Reaction. *Macromol. Chem. Phys.* **225**, 2300337 (2024).

[31] Franco, O. et al. Sensitizing TADF Absorption Using Variable Length Oligo(phenylene ethynylene) Antennae. *Front Chem* **8** (2020). 10.3389/fchem.2020.00126 PMC7054278 32175310

[32] Bohn, P., Frölich, M., Hahn, D., Schneider, R. V. & Meier, M. A. R. Uniform Macromolecules: Performance of Common Analytic Instruments in Detecting Impurities. *Macromol. Chem. Phys.* **226**, 2400396 (2025).

[33] Frisch, M. J. et al. Gaussian 16 Revision C.01 (2016).

[34] Adamo, C. & Barone, V. Toward reliable density functional methods without adjustable parameters: The PBE0 model. *J. Chem. Phys.* **110**, 6158–6170 (1999).

[35] Hariharan, P. C. & Pople, J. A. The influence of polarization functions on molecular orbital hydrogenation energies. *Theoret Chim. Acta*. **28**, 213–222 (1973).

[36] Francl, M. M. et al. Self-consistent molecular orbital methods. XXIII. A polarization‐type basis set for second‐row elements. *J. Chem. Phys.* **77**, 3654–3665 (1982).

[37] Grimme, S., Antony, J., Ehrlich, S. & Krieg, H. A consistent and accurate ab initio parametrization of density functional dispersion correction (DFT-D) for the 94 elements H-Pu. *J. Chem. Phys.* **132**, 154104 (2010). 20423165 10.1063/1.3382344

[38] Grimme, S., Ehrlich, S. & Goerigk, L. Effect of the damping function in dispersion corrected density functional theory. *J. Comput. Chem.* **32**, 1456–1465 (2011). 21370243 10.1002/jcc.21759

[39] Lu, T. & Chen, F. Multiwfn: A multifunctional wavefunction analyzer. *J. Comput. Chem.* **33**, 580–592 (2012). 22162017 10.1002/jcc.22885

[40] Liu, Z., Lu, T. & Chen, Q. An sp-hybridized all-carboatomic ring, cyclo[18]carbon: Electronic structure, electronic spectrum, and optical nonlinearity. *Carbon* **165**, 461–467 (2020).

[41] Zhang, K. et al. Solid-State Effect Induced Thermally Activated Delayed Fluorescence with Tunable Emission: A Multiscale Study. *J. Phys. Chem. A*. **124**, 8540–8550 (2020). 32966069 10.1021/acs.jpca.0c07152

[42] Wei, Z. et al. Thermally activated delayed fluorescence materials with aggregation-induced emission properties: a QM/MM study. *Phys. Chem. Chem. Phys.* **23**, 25789–25796 (2021). 34766607 10.1039/d1cp04190h

[43] Jiang, S. et al. Carbonyl (CO)/N-based thermally activated delayed fluorescent materials with high efficiency and fast reverse intersystem crossing rate: a theoretical design and study. *New. J. Chem.* **47**, 7686–7693 (2023).

[44] Ha, J. M., Hur, S. H., Pathak, A., Jeong, J. E. & Woo, H. Y. Recent advances in organic luminescent materials with narrowband emission. *NPG Asia Mater.* **13**, 1–36 (2021).

[45] Santos, P. L., dos, Stachelek, P., Takeda, Y. & Pander, P. Recent advances in highly-efficient near infrared OLED emitters. *Mater. Chem. Front.* **8**, 1731–1766 (2024).

[46] Wu, X., Ni, S., Wang, C. H., Zhu, W. & Chou, P. T. Comprehensive Review on the Structural Diversity and Versatility of Multi-Resonance Fluorescence Emitters: Advance, Challenges, and Prospects toward OLEDs. *Chem. Rev.* **125**, 6685–6752 (2025). 40344420 10.1021/acs.chemrev.5c00021 PMC12291210

[47] Li, K. et al. Highly phosphorescent platinum(II) emitters: photophysics, materials and biological applications. *Chem. Sci.* **7**, 1653–1673 (2016). 30155012 10.1039/c5sc03766b PMC6090519

[48] Würth, C., Grabolle, M., Pauli, J., Spieles, M. & Resch-Genger, U. Comparison of Methods and Achievable Uncertainties for the Relative and Absolute Measurement of Photoluminescence Quantum Yields. *Anal. Chem.* **9**, 3431–3439 (2011). 10.1021/ac2000303 21473570

[49] A Standard Default Color Space for the Internet. - sRGB https://www.w3.org/Graphics/Color/sRGB.html (1996).

[50] International Telecommunication Union BT. https://www.itu.int/rec/R-REC-BT.2020-0-201208-S/en (2015).

