# Supplementary material for: Sequence-defined donor-acceptor-donor oligo(para-phenylene ethynylene)s with emission across the visible spectrum
Source: Sci Rep. 2026 Jun 30;16:19916. doi: 10.1038/s41598-026-59109-2 (PMC13319820; doi:10.1038/s41598-026-59109-2)
Supplement: Supplementary file 1 — Supplementary Material 1 [file 41598_2026_59109_MOESM1_ESM.pdf]

Supplementry Information for

Sequence-defined donor-acceptor-donor oligo(*para*-phenylene ethynylene) with emission across the visible spectrum

Qianyu Cai,[1] Lars Boller,[1] and Michael A. R. Meier[1,2,*]

[1] Institute of Organic Chemistry (IOC), Karlsruhe Institute of Technology (KIT), 76131 Karlsruhe, Germany
[2] Institute of Biological and Chemical Systems – Functional Molecular Systems (IBCS-FMS), Karlsruhe Institute of Technology (KIT), 76131 Karlsruhe, Germany

* Corresponding author. Email: m.a.r.meier@kit.edu

Table of contents

1 Materials **S1**

2 Instrumentations **S2**

3 Impurity analysis for OPE-b' **S4**

4 Synthetic procedures **S5**

5 Quantum-chemical calculations **S16**

5 References

1 Materials

The following chemicals were used as received: bis(triphenylphosphine)palladium(II) chloride ($Pd(PPh_3)_2Cl_2$, >99.99%, Sigma-Aldrich); 1-bromopropane (99%, Sigma-Aldrich); 9,10-dibromoanthracene (98%, Sigma-Aldrich); copper(I) iodide (CuI, 99.9%, abcr); 1,4-dibromo-2,5-difluorobenzene (98%, BLDpharm); 4,7-dibromobenzo[*c*][1,2,5]thiadiazole (98%, BLDpharm); 4,9-dibromonaphtho[2,3-*c*][1,2,5]thiadiazole (98%, BLDpharm); diisopropylamine (DIPA, >99.5%, Sigma-Aldrich); potassium hydroxide (KOH, for analysis, Bernd Kraft); sodium hydrogencarbonate ($NaHCO_3$, laboratory reagent grade, Fisher Scientific); sodium hydroxide (NaOH, for analysis, Bernd Kraft); sodium sulfate (Na_2SO_4, pure, Bernd Kraft); tetrahydrofuran (THF, anhydrous, ≥99.5%, Thermo Scientific Chemicals); tetrakis(triphenylphosphine)palladium(0) ($Pd(PPh_3)_4$, 99%, Sigma-Aldrich); triethylamine (TEA, anhydrous, fluoro chem); trimethylsilylacetylene (98%, TCI); toluene (anhydrous, 99.85%, Thermo Scientific Chemicals); tris(dibenzylideneacetone)dipalladium(II) ($Pd_2(dba)_3$, >98%, fluoro chem); zinc bromide ($ZnBr_2$, 99%, abcr). Solvents like acetone, cyclohexane, dichloromethane, ethanol, ethyl acetate, methanol, *n*-pentane were used in HPLC grade without further purification. Flash column chromatography was performed using Silica gel (pore size 60 Å, 230-400 mesh particle size,

40-63 µm particle size, Sigma-Aldrich), Celite® 545 (Thermo Scientific Chemicals), and quartz sand (glowed and purified with hydrochloric acid, abcr).

2 Instrumentations

Nuclear magnetic resonance (NMR)

^{1}H and ^{13}C NMR spectra were recorded at the Karlsruhe Institute of Technology (KIT, Germany) on a Bruker Avance 400 NMR instrument at 400 MHz for ^{1}H NMR and 101 MHz for ^{13}C NMR or on a Bruker AVANCE DRX at 500 MHz for ^{1}H NMR and 126 MHz for ^{13}C NMR. $CDCl_3$ was used as solvent. Chemical shifts are presented in parts per million (δ) relative to residual solvent signals: δ = 7.26 ppm (^{1}H, $CDCl_3$) and δ = 77.16 ppm (^{13}C, $CDCl_3$). The spin multiplicity and corresponding signal patterns were abbreviated as follows: s = singlet, d = doublet, t = triplet, q = quartet, quint = quintet, sext = sextet, m = multiplet, dd = doublet of doublets, and br = broad signal. Coupling constants (J) are reported in Hertz (Hz). All measurements were recorded in a standard fashion at 25 °C unless otherwise stated. Full assignment of structures was aided by 2D NMR analysis (COSY, HSQC and HMBC).

Orbitrap electrospray-ionization mass spectrometry (ESI-MS)

Mass spectra were recorded on a Q Exactive (Orbitrap) mass spectrometer (Thermo Fisher Scientific, San Jose, CA, USA) equipped with an atmospheric pressure ionization source operating in the nebulizer assisted electrospray mode. The instrument was calibrated in the m/z-range 150-2000 using a standard containing caffeine, Met-Arg-Phe-Ala acetate (MRFA) and a mixture of fluorinated phosphazenes (Ultramark 1621, all from Sigma-Aldrich). A constant spray voltage of 3.5 kV, a dimensionless sheath gas of 6, and a sweep gas flow rate of 2 were applied. The capillary voltage and the S-lens RF level were set to 68.0 V and 320 °C, respectively.

Preparative thin layer chromatography (prep-TLC)

Prep-TLC was performed on glass-backed silica gel 60 F_{254} plates (20 × 20 cm, 2 mm thickness, MACHEREY-NAGEL). The crude material was applied as a narrow band approximately 3 cm from the bottom edge of the plate. Plates were developed using a mixture of cyclohexane/dichloromethane as the eluent. The eluent composition was optimized based on analytical TLC to achieve sufficient separation between the target product and impurities. After development, the product band was visualized under UV light (254 nm), scraped off, and taken off with dichloromethane (3 × 50 mL). The combined extracts were filtered and concentrated under reduced pressure to afford the purified compound.

Flash column chromatography

Flash column chromatography was performed on silica gel (see description above) as the stationary phase packed using the chosen eluent under positive pressure. The crude products were loaded onto the column as a concentrated solution in a minimal volume of eluent or adsorbed onto Celite® for dry loading.

Size-exclusion chromatography (SEC)

The SEC measurements were performed on a Shimadzu system equipped with an isocratic pump (LC-20AD), a refractive index detector (RID-20A, 24 °C), an autosampler (SIL-20A), and a Varian column oven (510, 50 °C). Separation was achieved using a three-column setup consisting of one SDV 3 µm, 8 × 50 mm precolumn and two SDV 3 µm, 1000 Å, 3 × 300 mm columns (PSS, Germany). Tetrahydrofuran (THF) stabilized with 250 ppm butylated hydroxytoluene (BHT, ≥99.9%, Sigma-Aldrich) was employed as the eluent at a flow rate of 1.0 mL min^{-1}. Calibration was performed with eight narrow polymethyl methacrylate (PMMA) standards ranging from 102 to 58 300 Da.

Infrared spectroscopy (IR)

The infrared spectra were recorded on a Bruker Alpha-p spectrometer equipped with attenuated total reflection (ATR) technology in the range of 4000 – 500 cm^{-1}, using 24 scans per measurement at a resolution of 4 cm^{-1}.

Ultraviolet–visible spectroscopy (UV–Vis)

The UV-Vis spectra were recorded at 20 °C using a Cary 3500 UV-Vis spectrophotometer equipped with a multicell Peltier temperature controller. All spectra were background-corrected against the corresponding solvent baseline.

Photoluminescence spectroscopy

The photoluminescence spectra were recorded on a Jobin-Yvon Fluoromax-4 fluorimeter equipped with a Peltier element (LFI3751) at 20 °C. Spectra were corrected for Raman scattering of the pure solvent. Calibration was performed using the Raman peak of water.

Quantum yield determination

The absolute quantum yields (Φ_{PL}) were determined in dilute, non-degassed toluene solution using a Quantaurus QY C11347 spectrometer (Hamamatsu). The excitation wavelength was selected based absorption maxima.

3 Impurity analysis for OPE-b'

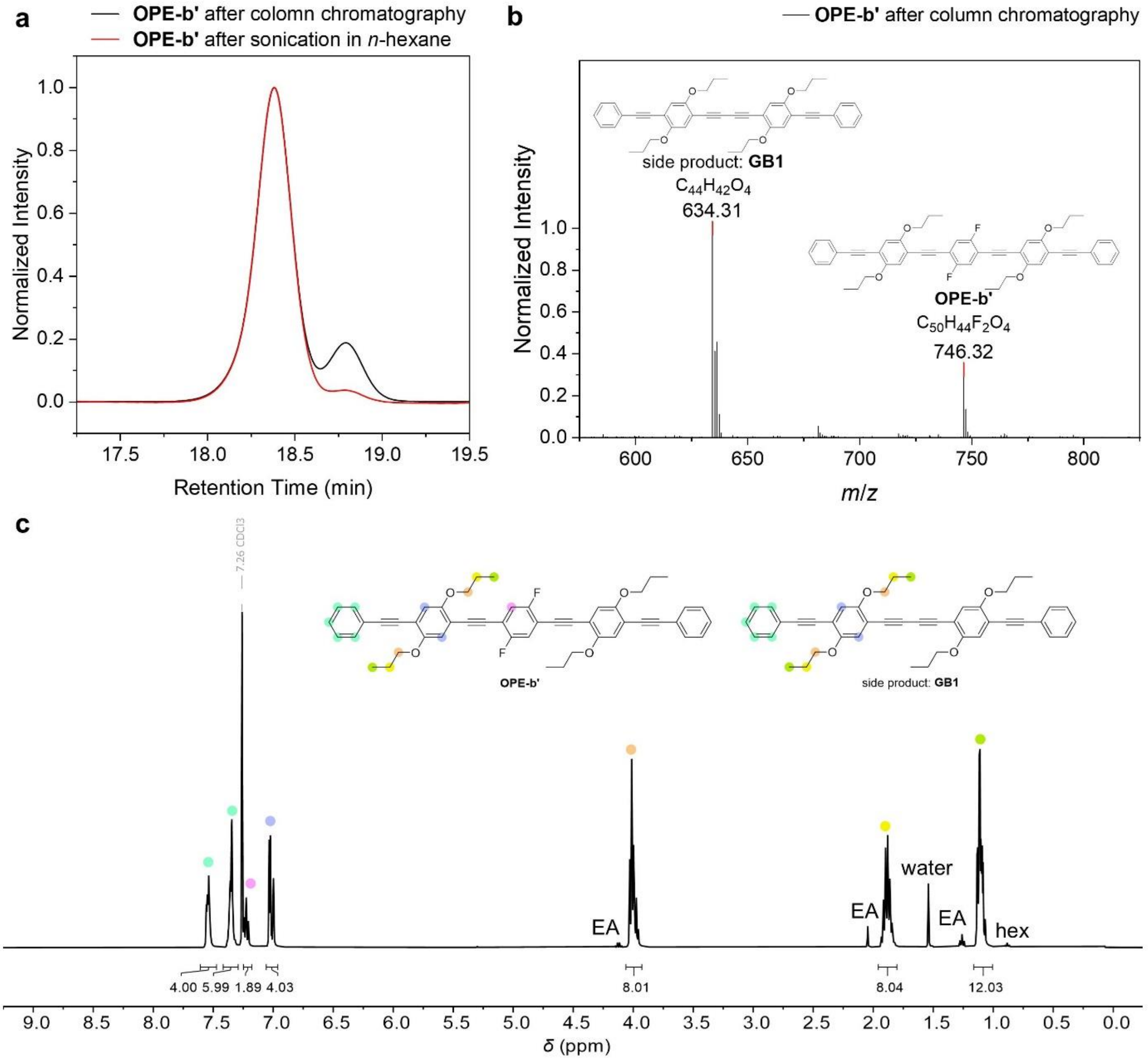

Figure S1. a) SEC analysis of **OPE-b'** after flash column chromatography (black) and subsequent purification by sonication in n-hexane (red), showing the removal of impurities of lower molecular weight. b) ESI-MS spectrum of chromatographically purified **OPE-b'**, indicating the presence of a Glaser-type homocoupling side product **GB1**. c) ^{1}H NMR spectrum of chromatographically purified **OPE-b'** in $CDCl_3$. Identified impurities: ethyl acetate (EA) and *n*-hexane (hex).

4 Synthetic procedures

2,2'-((2,5-Difluoro-1,4-phenylene)bis(ethyne-2,1-diyl))bis(1,4-dipropoxybenzene) (OPE-b)

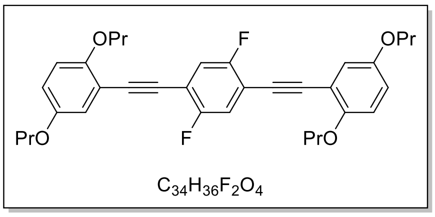

1,4-Dibromo-2,5-difluorobenzene (109 mg, 400 µmol, 1.00 equiv.), **B0** (218 mg, 1.00 mmol, 2.50 equiv.), copper(**I**) iodide (3.8 mg, 20 µmol, 0.05 equiv.), and $Pd(PPh_3)_2Cl_2$ (8.4 mg, 12 µmol, 0.03 equiv.) were degassed and added to 2.00 mL anhydrous THF and 1.12 mL anhydrous triethylamine under argon. After 18 h stirring at room temperature, complete conversion was indicated by TLC. The volatiles were removed under reduced pressure and the residue was purified by column chromatography (cyclohexane → cyclohexane/ethyl acetate 30:1) to yield a white solid (62%).

TLC (cyclohexane/ethylacetate 20:1): R_f = 0.45.

^{1}H NMR (500 MHz, $CDCl_3$): δ (ppm) = 7.21 (t, J = 7.4 Hz, 2H, H_{ar}), 7.03 (d, J = 2.9 Hz, 2H, H_{ar}), 6.88 (dd, J = 9.0 Hz, J = 3.0 Hz, 2H, H_{ar}), 6.83 (d, J = 9.0 Hz, 2H, H_{ar}), 3.98 (t, J = 6.4 Hz, 4H, OCH_2), 3.89 (t, J = 6.6 Hz, 4H, OCH_2), 1.91–1.75 (m, 8H, $CH_2CH_2CH_3$), 1.09 (t, J = 7.4 Hz, 6H, CH_3), 1.03 (t, J = 7.4 Hz, 6H, CH_3).

^{13}C NMR (126 MHz, $CDCl_3$): δ (ppm) = 158.2 (dd, J = 249.8 Hz, J = 3.6 Hz, C_qF), 154.5 (C_qO), 152.9 (C_qO), 119.5–119.1 (m, CH), 118.6 (CH), 117.7 (CH), 114.2 (2C, CH), 113.6–113.4 (m, C_q), 112.7 (CH), 93.9 (C_q), 85.7 (C_q), 71.4 (OCH_2), 70.4 (OCH_2), 22.8 (CH_2), 10.7 (CH_3).

HRMS (ASAP, m/z): $[M+H]^+$ calcd for $C_{34}H_{36}F_2O_4$ 547.2654, found 547.2653.

IR (ATR): $\tilde{\nu}$ (cm^{-1}) = 2960 (w, C-H_{sp3}), 2929 (w, C-H_{sp3}), 2876 (w, C-H_{sp3}), 2217 (w, C≡C), 1600 (w, C=C_{ar}), 1493 (vs), 1388 (s), 1269 (vs), 1212 (vs), 1140 (vs), 1065 (vs), 991 (vs), 973 (vs), 870 (vs), 845 (vs), 812 (vs).

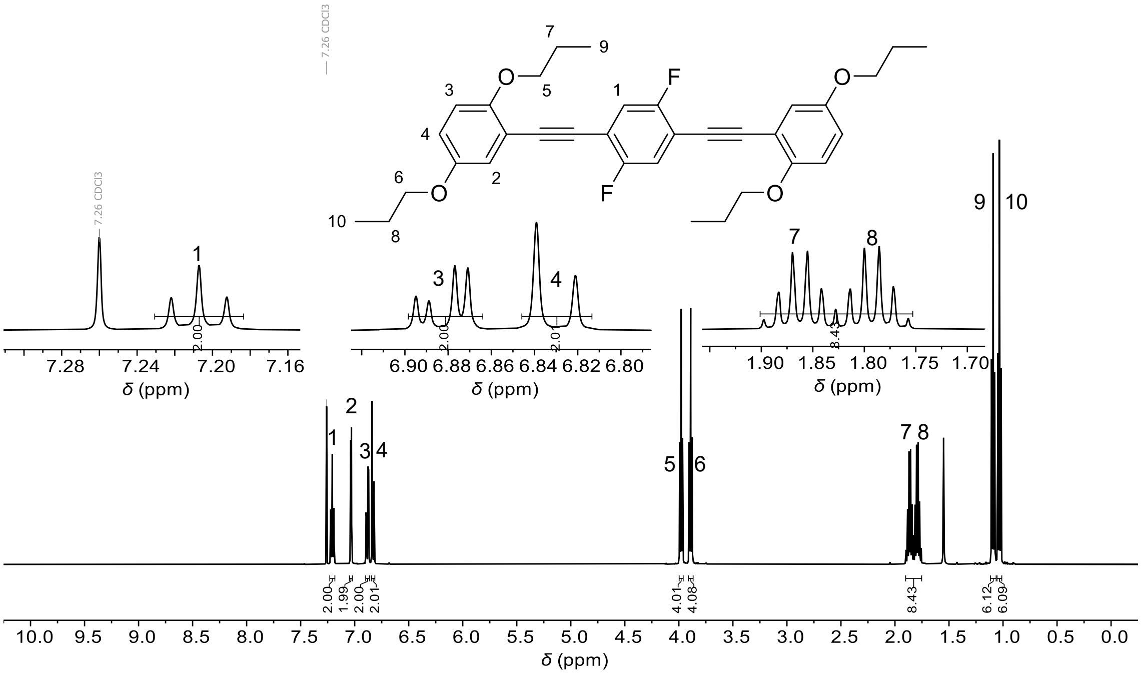

Figure S2. ^{1}H NMR spectrum of compound **OPE-b**, measured in $CDCl_3$ at 500 MHz.

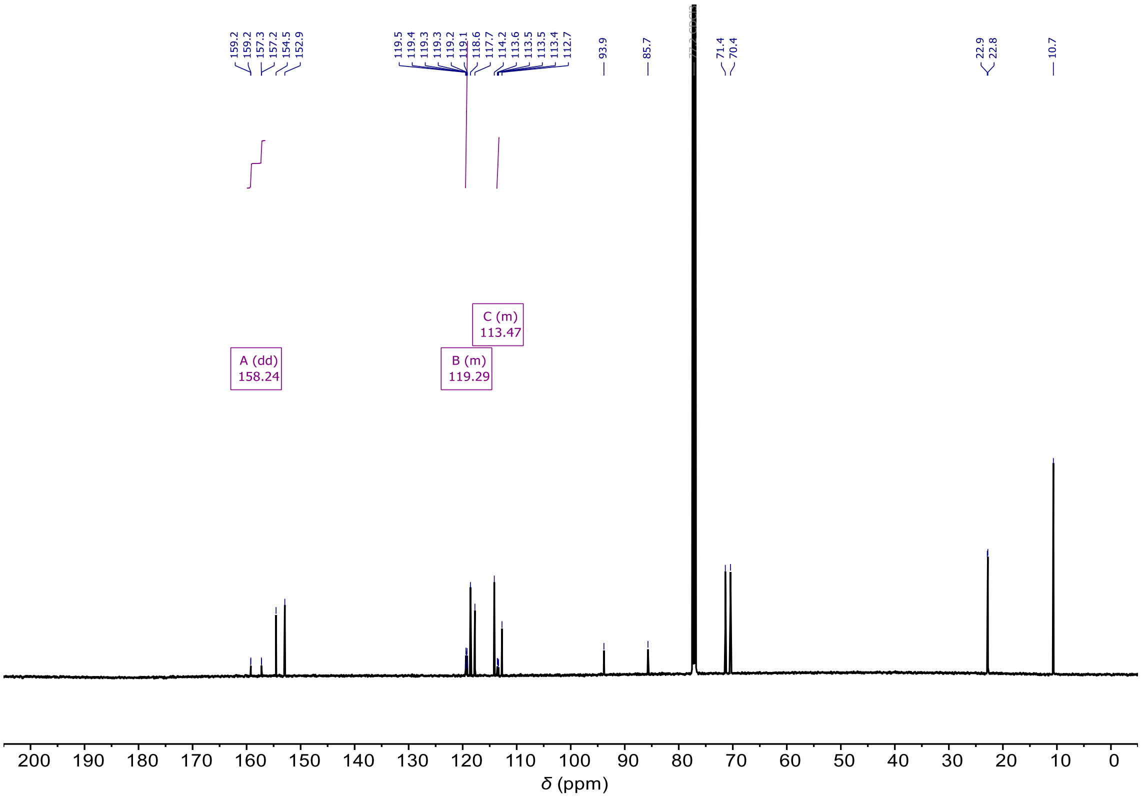

Figure S3. ^{13}C NMR spectrum of compound **OPE-b**, measured in $CDCl_3$ at 126 MHz.

9,10-Bis((2,5-dipropoxyphenyl)ethynyl)anthracene (OPE-g)

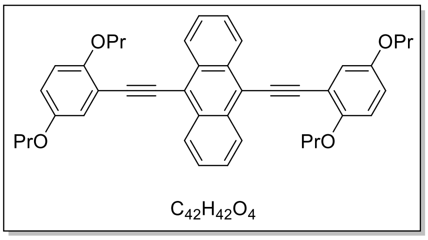

9,10-Dibromanthracene (168 mg, 500 µmol, 1.00 equiv.), Pd_2dba_3 (4.8 mg, 5.0 µmol, 0.01 equiv.), triphenylphosphine (3.9 mg, 15 µmol, 0.03 equiv.), and copper(I) iodide (2.9 mg, 15 µmol, 0.03 equiv.) were degassed and suspended under continuous argon flow in 10 mL anhydrous THF and 1.4 mL anhydrous triethylamine (1.0 g, 10 mmol, 20 equiv.). After flushing with argon for 10 min, **B0** (240 mg, 1.10 mmol, 2.20 equiv.) was added dropwise, and the reaction mixture was stirred at 65 °C for 20 h. The reaction mixture was cooled down to room temperature and poured onto a short silica gel plug (5 cm) and washed with cyclohexane. Purification by flash column chromatography (cyclohexane → cyclohexane/DCM 1:2) yielded the title compound as an orange solid (78.2 mg, 138 µmol, 69%).

TLC (cyclohexane/DCM 3:2): R_f = 0.35.

^{1}H NMR (400 MHz, $CDCl_3$): δ (ppm) = 8.84 (m, 4H, H_{ar}), 7.62 (m, 4H, H_{ar}), 7.30–7.24 (m, 2H, H_{ar}), 6.95–6.88 (m, 4H, H_{ar}), 4.10 (t, J = 6.5 Hz, 4H, OCH_2), 3.98 (t, J = 6.5 Hz, 4H, OCH_2), 2.06 (h, J = 7.1 Hz, 4H, $CH_2CH_2CH_3$), 1.85 (h, J = 7.1 Hz, 4H, $CH_2CH_2CH_3$), 1.18 (t, J = 7.4 Hz, 6H, CH_3), 1.08 (t, J = 7.4 Hz, 6H, CH_3).

^{13}C NMR (101 MHz, $CDCl_3$): δ (ppm) = 154.5 (C_qO), 152.9 (C_qO), 132.2 (C_q), 127.7 (CH), 126.7 (CH), 118.7 (CH), 116.8 (CH), 113.7 (C_q), 113.0 (CH), 99.4 (C_q), 91.0 (C_q), 71.0 (OCH_2), 70.5 (OCH_2), 23.1 (CH_2), 22.9 (CH_2), 11.0 (CH_3), 10.7 (CH_3).

HRMS (ASAP, m/z): $[M]^+$ calcd for $C_{42}H_{42}O_4$ 610.3078, found 610.3071.

IR (ATR): $\tilde{\nu}$ (cm^{-1}) = 2966 (w, C-H_{sp3}), 2935 (w, C-H_{sp3}), 2874 (w, C-H_{sp3}), 2193 (vw, C≡C), 1600 (w, C=C_{ar}), 1497 (s), 1467 (m), 1434 (m), 1393 (w), 1296 (w), 1267 (s), 1226 (vs), 1193 (s), 1160 (m), 1121 (m), 1041 (vs), 1020 (vs), 850 (s), 812 (s), 761 (vs), 638 (s).

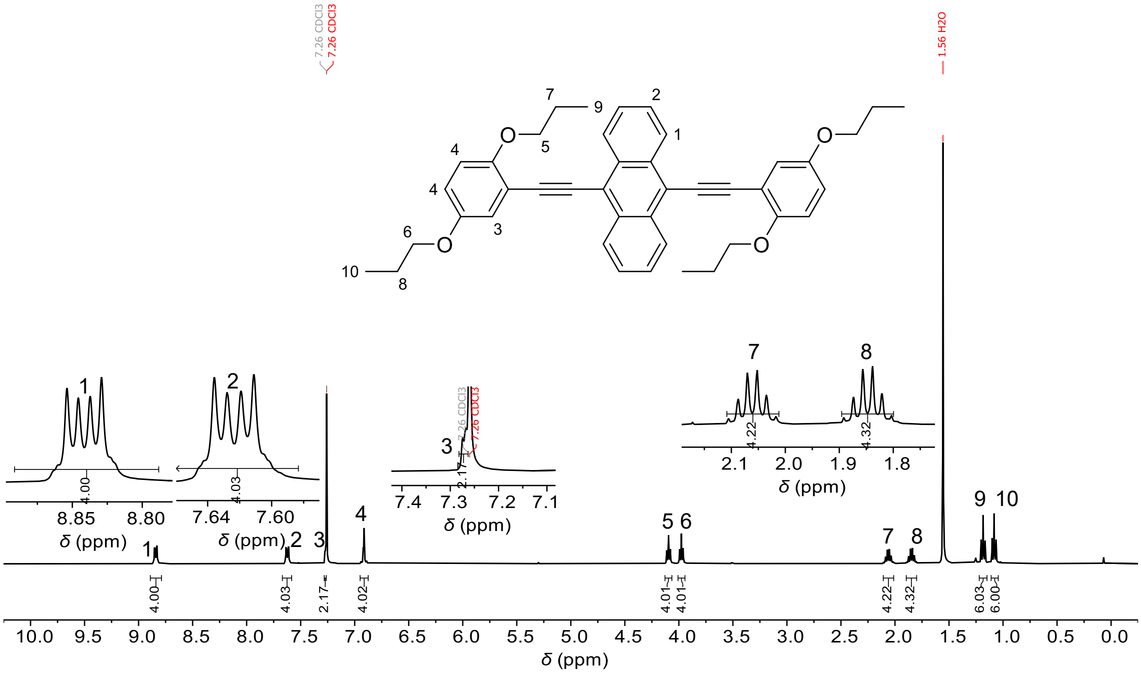

Figure S4. ^{1}H NMR spectrum of **OPE-g**, measured in $CDCl_3$ at 400 MHz.

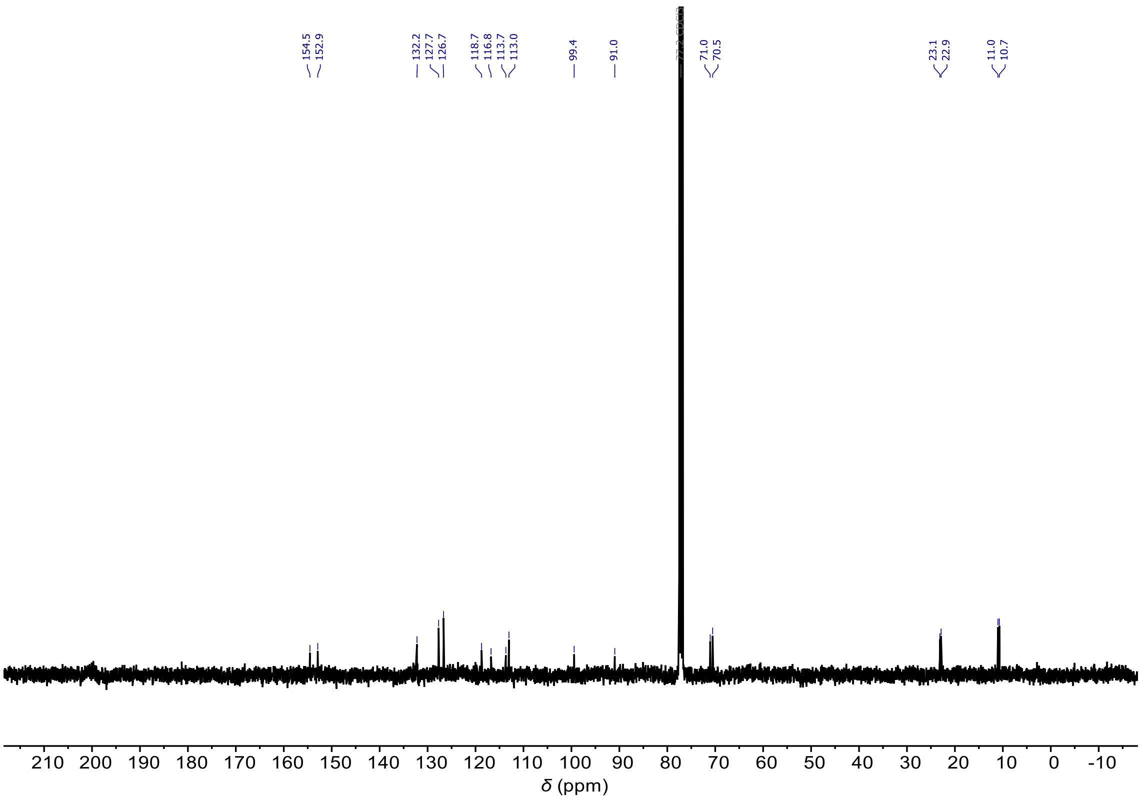

Figure S5. ^{13}C NMR spectrum of **OPE-g**, measured in $CDCl_3$ at 101 MHz.

4,7-Bis((2,5-dipropoxyphenyl)ethynyl)benzo[*c*][1,2,5]thiadiazole (OPE-y)

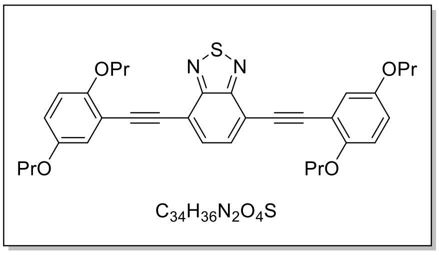

4,7-Dibromobenzo[*c*][1,2,5]thiadiazole (96.0 mg, 440 µmol, 1.00 equiv.), **B0** (96.0 mg, 440 µmol, 2.21 equiv.), $Pd(PPh_3)_4$ (9.9 mg, 8.6 µmol, 0.04 equiv.), and copper(**I**) iodide (3.8 mg, 20 µmol, 0.10 equiv.) were degassed and suspended under continuous argon flow in 2.0 mL anhydrous THF and 1.4 mL anhydrous diisopropylamine (405 mg, 3.96 mmol, 19.9 equiv.). The reaction mixture was stirred at room temperature for 20 h. After a complete conversion confirmed by TLC, the reaction mixture was poured onto a short silica gel plug (5 cm) and washed with cyclohexane. Purification by flash column chromatography (cyclohexane → cyclohexane/DCM 1:2) yielded the title compound as an orange solid (78.2 mg, 138 µmol, 69%).

TLC (cyclohexane/ethyl acetate 5:1): R_f = 0.42.

^{1}H NMR (400 MHz, $CDCl_3$): *δ* (ppm) = 7.76 (s, 2H, H_{ar}), 7.15 (d, *J* = 2.9 Hz, 2H, H_{ar}), 6.90 (dd, *J* = 9.0 Hz, *J* = 2.9 Hz, 2H, H_{ar}), 6.86 (dd, 2H, H_{ar}), 4.03 (t, *J* = 6.5 Hz, 4H, OCH_2), 3.91 (t, *J* = 6.6 Hz, 4H, OCH_2), 1.92 (h, *J* = 7.3 Hz, 4H, $CH_2CH_2CH_3$), 1.80 (h, *J* = 7.2 Hz, 4H, $CH_2CH_2CH_3$), 1.12 (t, *J* = 7.4 Hz, 6H, CH_3), 1.04 (t, *J* = 7.4 Hz, 6H, CH_3).

^{13}C NMR (101 MHz, $CDCl_3$): *δ* (ppm) = 154.6 (C_q), 154.5 (C_q), 152.8 (C_q), 132.2 (*C*H), 118.5 (*C*H), 117.7 (*C*H), 117.4 (C_q), 114.1 (*C*H), 112.9 (C_q), 94.3 (C_q), 89.3 (C_q), 71.3 (OCH_2), 70.3 (OCH_2), 22.8 (CH_2), 22.7 (CH_2), 10.7 (CH_3), 10.5 (CH_3).

HRMS (ESI, *m*/*z*): $[M+H]^+$ calcd for $C_{34}H_{36}N_2O_4S$ 569.2469, found 569.2471.

IR (ATR): $\tilde{\nu}$ (cm^{-1}) = 2960 (m, C-H_{sp3}), 2931 (m, C-H_{sp3}), 2874 (m, C-H_{sp3}), 2205 (w, C≡C), 1600 (w, C=C_{ar}), 1500 (vs), 1462 (s), 1393 (m), 1271 (vs), 1214 (vs), 1140 (s), 1119 (s), 1070 (s), 1049 (m), 1020 (s), 985 (vs), 975 (vs), 843 (vs), 802 (vs), 736 (m), 634 (m), 541 (s), 508 (s).

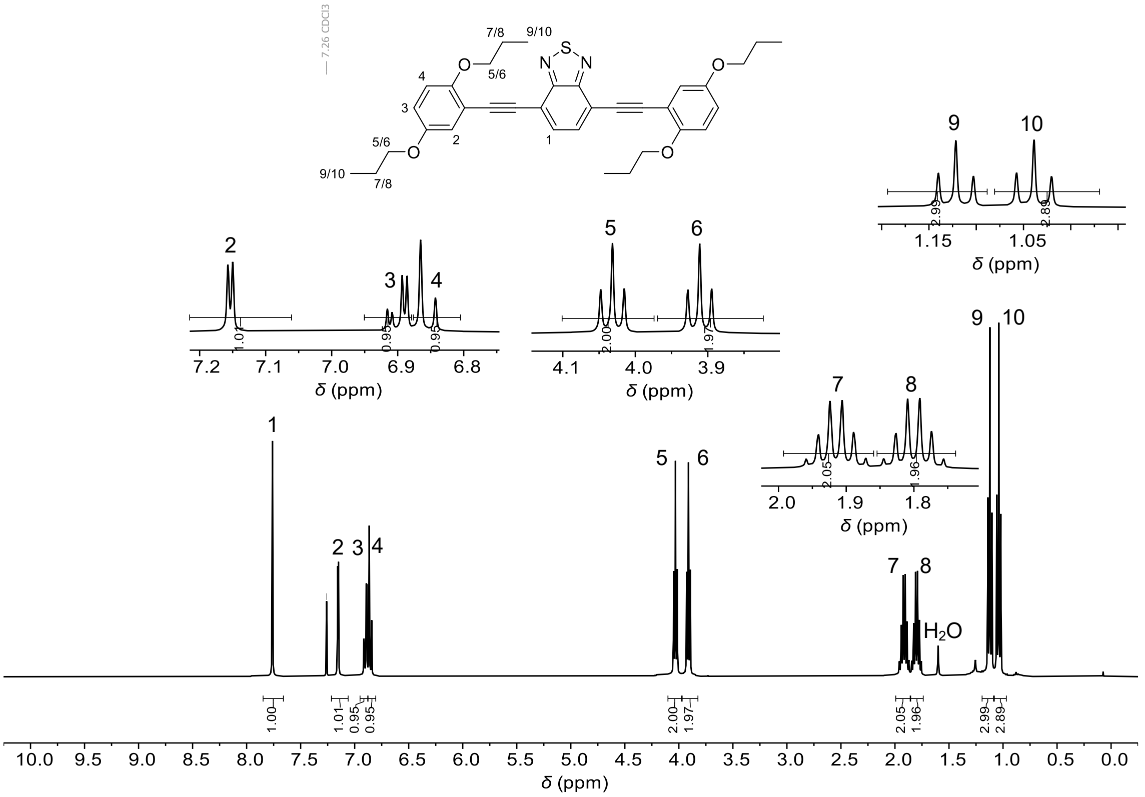

Figure S6. ^{1}H NMR spectrum of **OPE-y**, measured in $CDCl_3$ at 400 MHz.

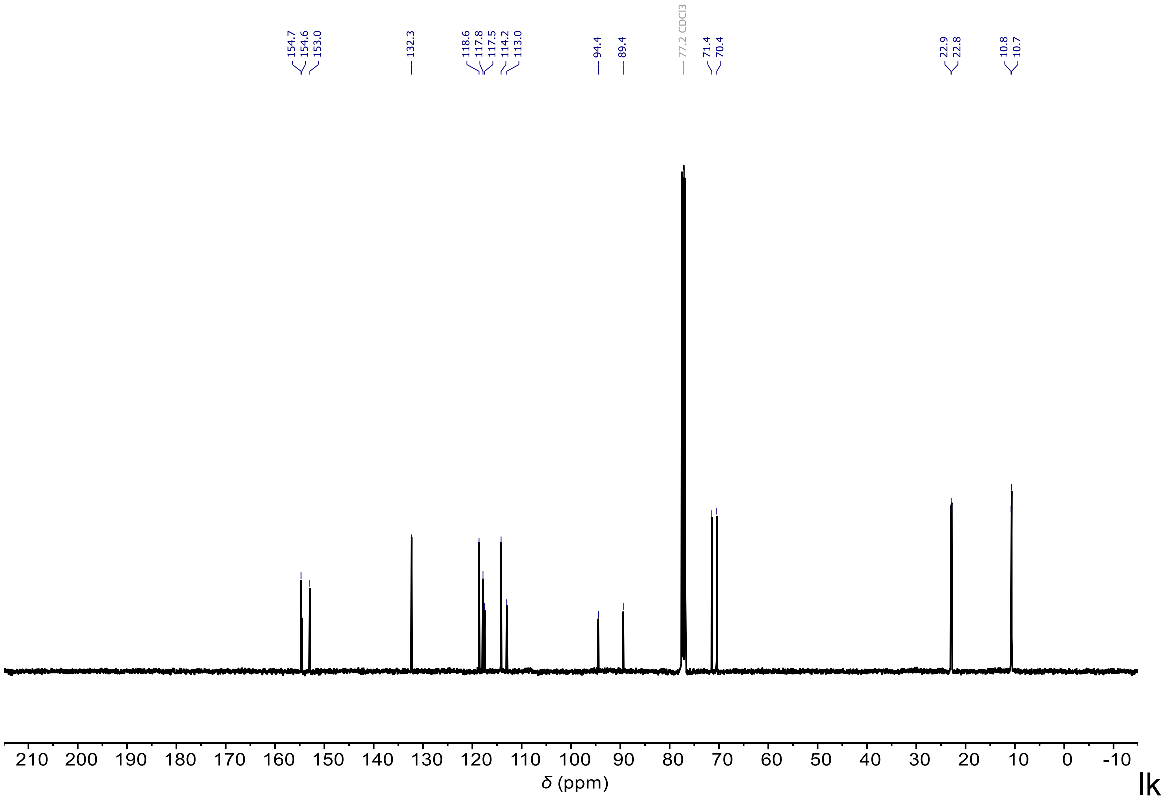

lk

Figure S7. ^{13}C NMR spectrum of **OPE-y**, measured in $CDCl_3$ at 101 MHz.

4,9-Bis((2,5-dipropoxyphenyl)ethynyl)naphtho[2,3-*c*][1,2,5]thiadiazole (OPE-r)

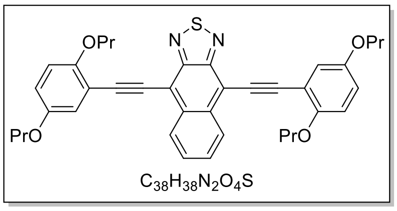

4,9-Dibromonaphtho[2,3-*c*][1,2,5]thiadiazole (50.0 mg, 145 µmol, 1.00 equiv.), **B0** (95.2 mg, 436 µmol, 3.00 equiv.), $Pd_2(dba)_3$ (1.3 mg, 1.5 µmol, 0.01 equiv.), triphenylphosphine (1.1 mg, 4.4 µmol, 0.03 equiv.) and copper(I) iodide (1.4 mg, 7.3 µmol, 0.05 equiv.) were degassed and suspended under continuous argon flow in 3.0 mL anhydrous THF and 0.41 mL anhydrous triethylamine (0.29 g, 2.9 mmol, 20 equiv.). Under argon atmosphere, the reaction mixture was stirred for 20 h at 65 °C. After a complete conversion was confirmed by TLC, the reaction mixture was poured onto a short silica gel plug (5 cm) and washed with cyclohexane. Purification by flash column chromatography (cyclohexane → cyclohexane/DCM 1:2) and subsequent washing with methanol yielded the title compound as an orange solid (74.8 mg, 121 µmol, 83%).

TLC (cyclohexane/DCM 3:2): R_f = 0.55.

^{1}H NMR (400 MHz, $CDCl_3$): δ = 8.85–8.80 (m, 2H, H_{ar}), 7.58–7.54 (m, 2H, H_{ar}), 7.29 (d, J = 2.8 Hz, 2H, H_{ar}), 6.94 (dd, J = 9.0 Hz, J = 3.0 Hz, 2H, H_{ar}), 6.89 (d, J = 9.0 Hz, 2H, H_{ar}), 4.09 (t, J = 6.5 Hz, 4H, OCH_2), 3.95 (t, J = 6.6 Hz, 4H, OCH_2), 2.03 (h, J = 7.3 Hz, 4H, $CH_2CH_2CH_3$), 1.83 (h, J = 7.3 Hz, 4H, $CH_2CH_2CH_3$), 1.17 (t, J = 7.4 Hz, 6H, CH_3), 1.06 (t, J = 7.4 Hz, 6H, CH_3).

^{13}C NMR (101 MHz, $CDCl_3$): δ = 154.7 (C_q), 152.8 (C_q), 152.5 (C_q), 134.9 (C_q), 128.1 (CH), 127.6 (CH), 118.4 (CH), 117.8 (CH), 113.2 (CH), 112.9 (C_q), 112.9 (C_q), 101.2 (C_q), 89.6 (C_q), 71.0 (OCH_2), 70.4 (OCH_2), 23.0 (CH_2), 22.7 (CH_2), 10.8 (CH_3), 10.6 (CH_3).

HRMS (ESI, *m*/*z*): $[M]^+$ calcd for $C_{38}H_{38}N_2O_4S$ 618.2547, found 618.2546.

IR (ATR): $\tilde{\nu}$ (cm^{-1}) = 2960 (m, C-H_{sp3}), 2871 (w, C-H_{sp3}), 2189 (vw, C≡C), 1600 (w, C=C_{ar}), 1497 (vs), 1456 (s), 1413 (w), 1390 (m),1265 (s), 1228 (vs), 1207 (vs, C-O), 1131 (m), 1119 (m), 1043 (s), 1024 (vs), 1010 (vs), 948 (m), 905 (m), 856 (vs), 796 (vs), 753 (vs), 738 (s), 522 (vs).

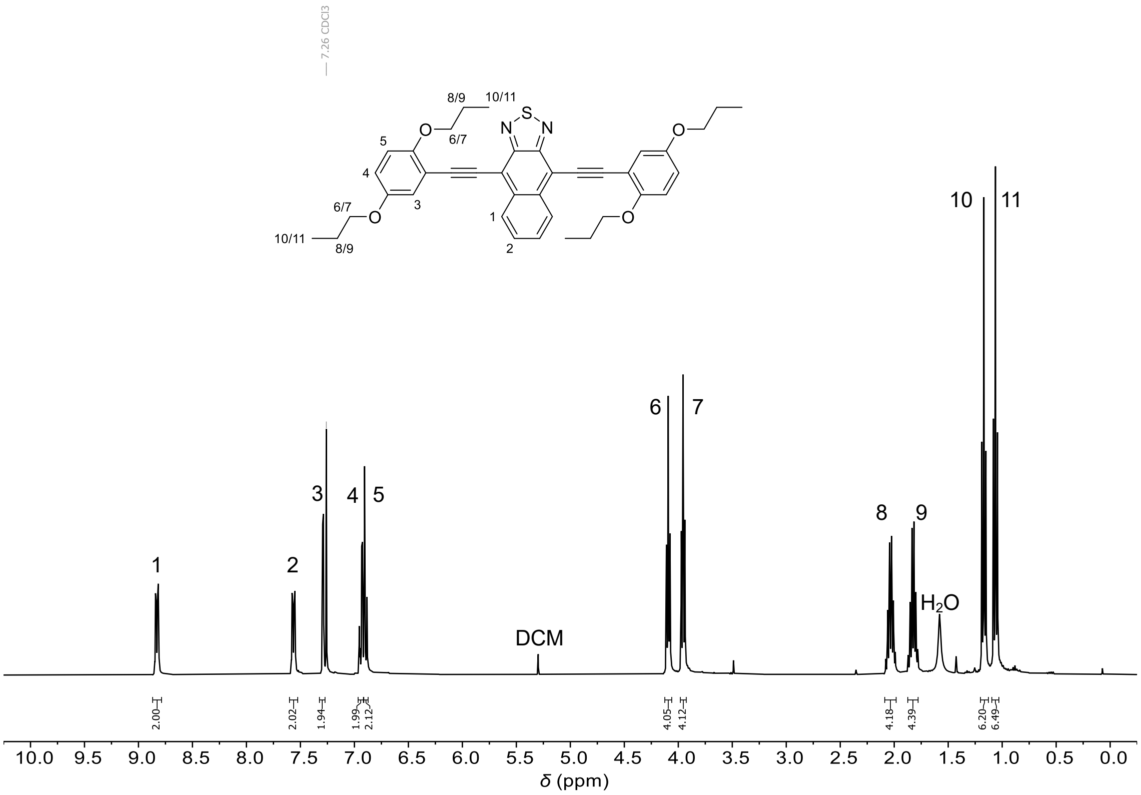

Figure S8. ^{1}H NMR spectrum of **OPE-r**, measured in $CDCl_3$ at 400 MHz.

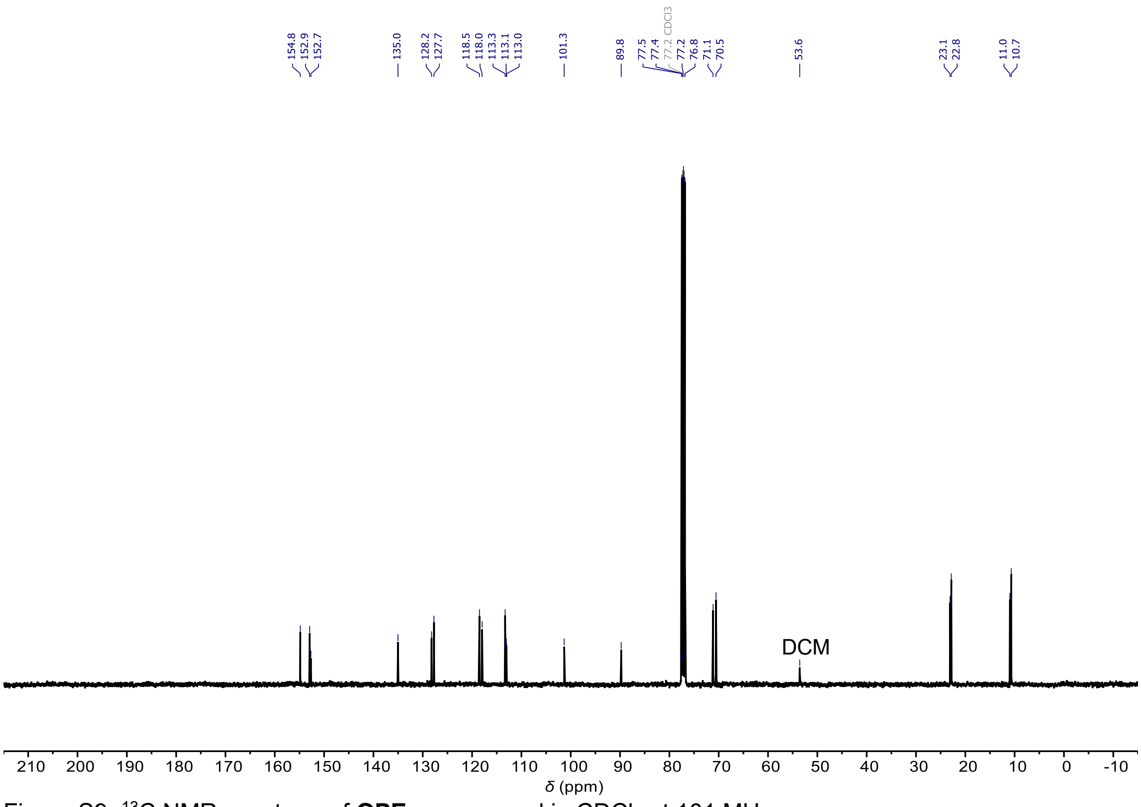

Figure S9. ^{13}C NMR spectrum of **OPE-r**, measured in $CDCl_3$ at 101 MHz.

5,5'-((2,5-Difluoro-1,4-phenylene)bis(ethyne-2,1-diyl))bis(2-(phenylethynyl)-1,4-dipropoxybenzene) (OPE-b')

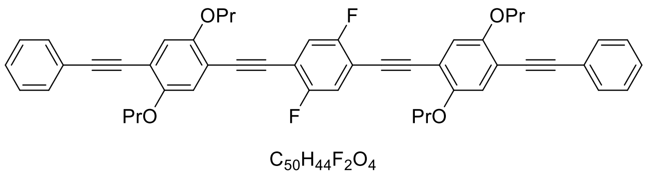

A mixture of **B1** (131 mg, 411 µmol, 2.06 equiv.), 1,4-dibromo-2,5-difluorobenzene (54.3 mg, 200 µmol, 1.00 equiv.), $Pd(PPh_3)_4$ (13.9 mg, 12.0 µmol, 0.06 equiv.) and copper(I) iodide (4.70 mg, 24.7 µmol, 0.12 equiv.) was degassed and then dissolved in 4 mL anhydrous toluene. After purging with argon for 5 minutes, 0.60 mL diisopropylamine (0.43 g, 4.3 mmol, 10 equiv.) were added. The reaction mixture was stirred under argon at room temperature for 23 h. The solvent was removed under reduced pressure. The residue was taken up with DCM, washed with water and brine. The organic phase was dried over Na_2SO_4 and concentrated under reduced pressure. The crude product was purified by flash column chromatography (cyclohexane to cyclohexane/ethyl acetate 20:1) and further by prep-TLC (cyclohexane/dichloromethane 3:1). The title compound was obtained after sonication in *n*-hexane as a neon-yellow solid (57.6 mg, 76.6 µmol, 38%).

TLC (cyclohexane/DCM 1:1): R_f = 0.32.

^{1}H NMR (400 MHz, $CDCl_3$): δ (ppm) = 7.56–7.53 (m, 4H, H_{ar}), 7.37–7.34 (m, 6H, H_{ar}), 7.23 (t, J = 7.6 Hz, 2H, FCCH_{ar}), 7.03–7.00 (m, 4H, H_{ar}), 4.03–3.96 (m, 8H, OCH_2), 1.93–1.84 (m, 8H, $CH_2CH_2CH_3$), 1.13–1.07 (m, 12H, CH_3).

^{13}C NMR (101 MHz, $CDCl_3$): δ (ppm) = 158.2 (dd, J = 249.8 Hz, J = 3.6 Hz, C_qF) 154.1 (C_qO), 153.6 (C_qO), 131.8 (CH), 128.5 (CH), 123.5 (C_q), 119.3–119.0 (m, CH), 117.9 (CH), 117.1 (CH), 117.0 (CH), 115.3 (C_q), 112.9 (C_q), 95.5 (C_q), 93.9 (C_q), 87.2 (C_q), 85.9 (C_q), 71.3 (OCH_2), 71.3 (OCH_2), 22.9 (CH_2), 22.8 (CH_2), 10.7 (CH_3), 10.6 (CH_3). Impurity signals at 155.2 and 153.6 ppm were assigned to phenolic C_q resonances of the side product **GB1**.

HRMS (ESI, *m/z*): M^+ calcd for $C_{50}H_{44}F_2O_4$ 746.3202, found 746.3167.

IR (ATR) $\tilde{\nu}$ (cm^{-1}) = 2962 (w, C-H_{sp3}), 2921 (w, C-H_{sp3}), 2874 (w, C-H_{sp3}), 2211 (vw, C≡C), 1596 (vw, C=C_{ar}), 1487 (s), 1469 (m), 1386 (s), 1275 (s), 1214 (vs), 1065 (m), 1016 (m), 985 (s), 870 (m), 858 (s), 751 (vs), 685 (vs), 527 (w).

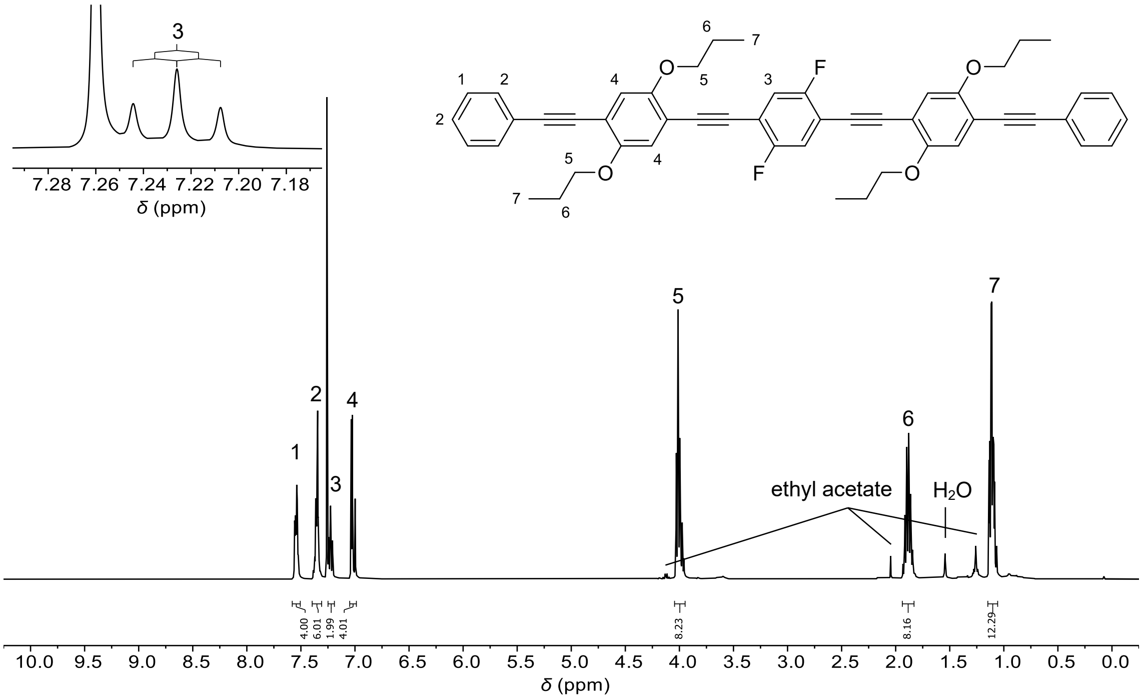

Figure S10. ^{1}H NMR spectrum of **OPE-b'**, measured in $CDCl_3$ at 400 MHz.

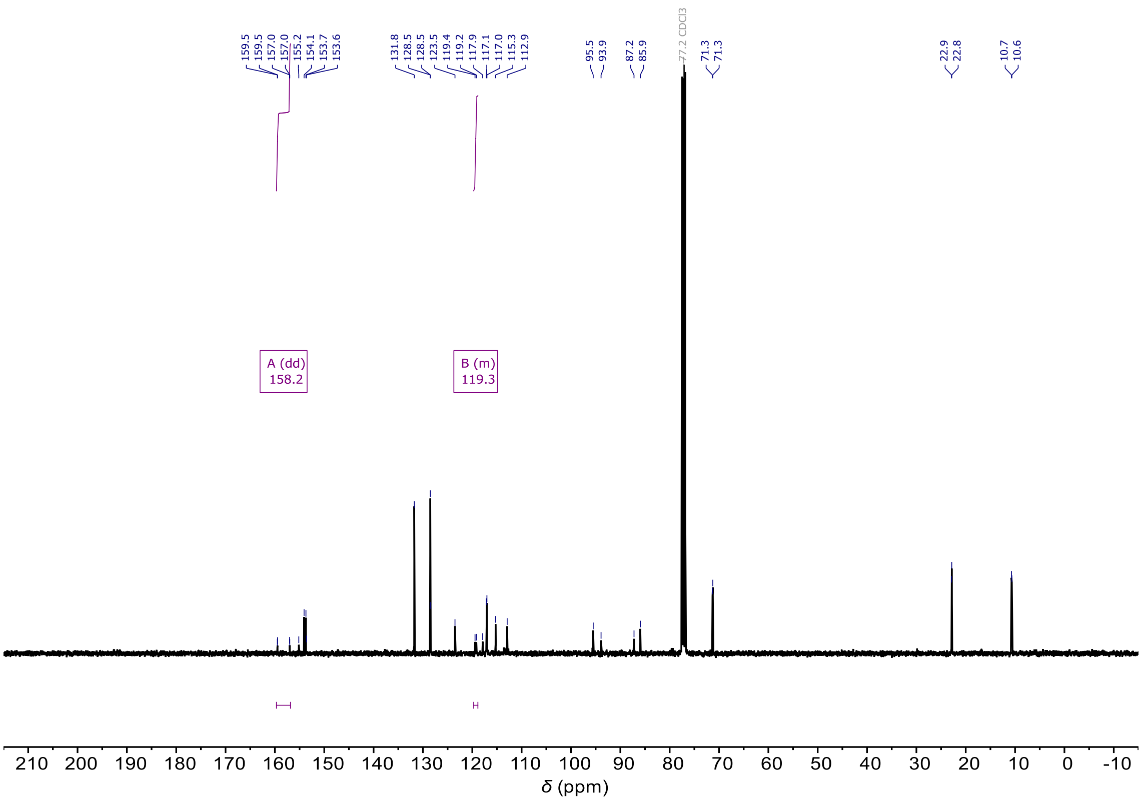

Figure S11. ^{13}C NMR spectrum of **OPE-b'**, measured in $CDCl_3$ at 101 MHz.

9,10-Bis((4-(phenylethynyl)-2,5-dipropoxyphenyl)ethynyl)anthracene (OPE-g')

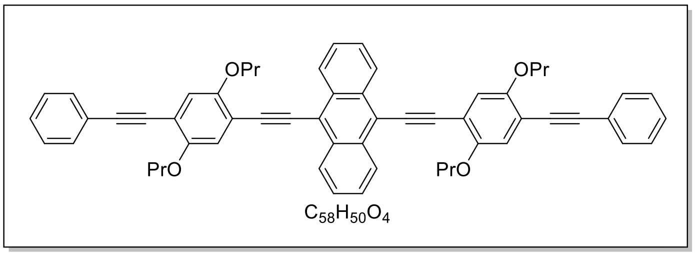

A mixture of **B1** (129 mg, 404 µmol, 2.02 equiv.), 9,10-dibromoanthracene (67.1 mg, 200 µmol, 1.00 equiv.), $Pd(PPh_3)_4$ (14.1 mg, 12.2 µmol, 0.06 equiv.) and copper(I) iodide (4.80 mg, 25.2 µmol, 0.13 equiv.) was degassed and added to 4.0 mL anhydrous toluene and 0.60 mL diisopropylamine (0.43 g, 4.3 mmol, 11 equiv.). The reaction mixture was stirred under argon at 21 °C for 3 days. After complete conversion was confirmed by TLC, the reaction mixture was poured onto a short silica gel plug (5 cm), washed with cyclohexane, and eluted with DCM. The crude product was subsequently washed with methanol and cyclohexane. After removal of the volatiles under reduced pressure, the title compound was obtained as an orange solid (35.8 mg, 44.1 µmol 22%).

TLC (cyclohexane/DCM 1:1): $R_f = 0.24$.

^{1}H NMR spectra was attempted in several deuterated solvents ($CDCl_3$, C_6D_6 and THF-d_8). However, well-resolved spectra could not obtained due to insufficient solubility. The following assignment is based on measurements in THF-d_8 at 500 MHz: δ (ppm) = 8.90 (dd, J = 6.6, 3.3 Hz, 4H, H_{ar}), 7.67 (dd, J = 6.7, 3.3 Hz, 4H, H_{ar}), 7.59–7.49 (m, 4H, H_{ar}), 7.43–7.31 (m, 8H, H_{ar}), 7.20 (s, 2H, H_{ar}), 4.17 (t, J = 6.5 Hz, 4H, OCH_2), 4.12 (t, J = 6.3 Hz, 4H, OCH_2), 2.15–1.99 (m, 8H, $CH_2CH_2CH_3$), 1.21 (t, J = 7.5 Hz, 6H, CH_3), 1.16 (t, J = 7.4 Hz, 6H, CH_3).

^{13}C NMR: not available; spectra suffered from poor resolution under the conditions tested.

HRMS (ESI, m/z): $[M+H]^+$ calcd for $C_{58}H_{50}O_4$ 811.3782; found 811.3743.

IR (ATR): $\tilde{\nu}$ (cm^{-1}) = 2964 (w, C-H_{sp3}), 2904 (w, C-H_{sp3}), 2874 (w, C-H_{sp3}), 1504 (s, C=C_{ar}), 1425 (m), 1386 (s), 1273 (m), 1216 (vs), 1041 (m), 1022 (vs), 905 (w), 860 (s), 775 (w), 761 (vs), 691 (m), 640 (s), 613 (w), 531 (w).

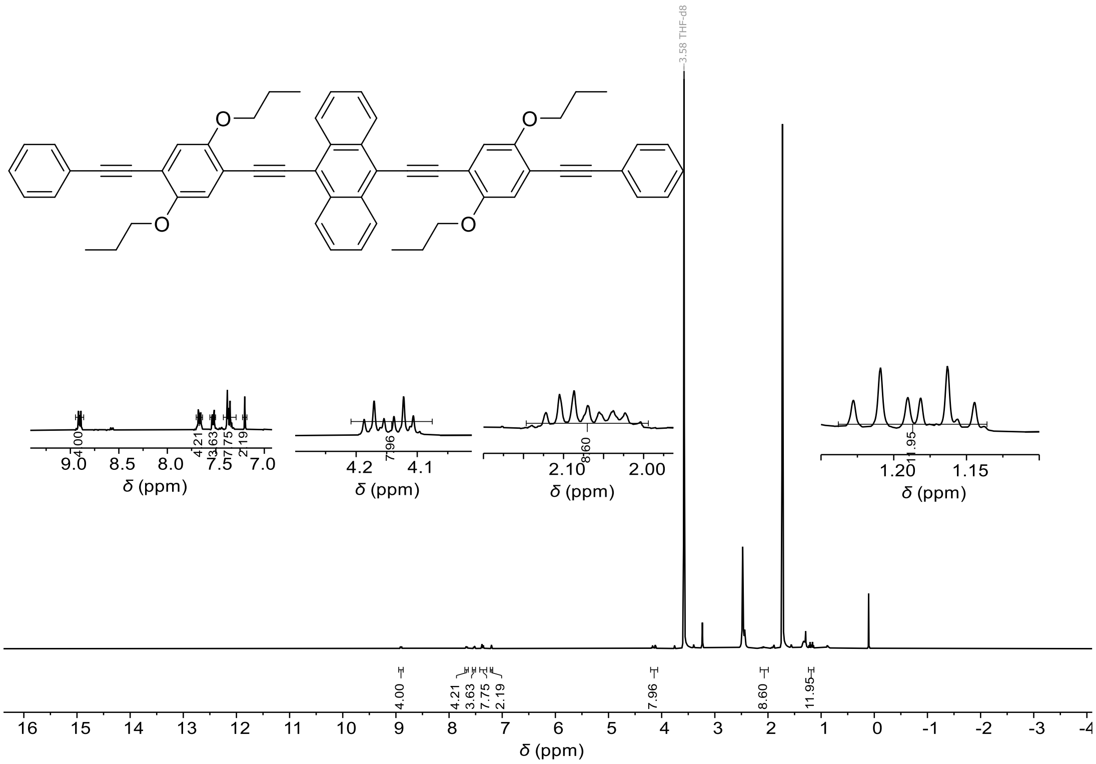

Figure S12. ^{1}H NMR spectrum of **OPE-g'**, measured in THF-d_8 at 500 MHz.

4,7-Bis((4-(phenylethynyl)-2,5-dipropoxyphenyl)ethynyl)benzo[*c*][1,2,5]thiadiazole (OPE-y')

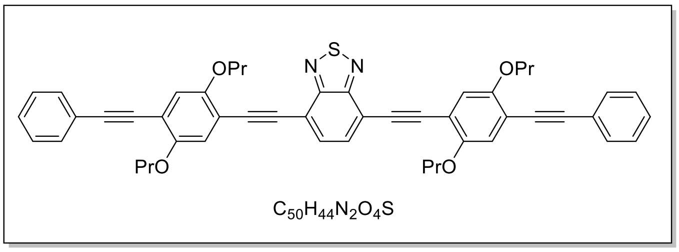

A mixture of **B1** (124 mg, 390 µmol, 2,05 equiv.), 1,4-dibromo-2,5-difluorobenzene (55.9 mg, 190 µmol, 1.00 equiv.), $Pd(PPh_3)_4$ (13.9 mg, 12.0 µmol, 0.06 equiv.) and copper(I) iodide (4.57mg, 24.0 µmol, 0.13 equiv.) was degassed and added to 4.0 mL anhydrous toluene and 0.60 mL diisopropylamine (0.43 g, 4.3 mmol, 11 equiv.). After stirring at room temperature for 2 days, complete conversion was indicated by TLC. The reaction mixture was poured onto a short silica gel plug (5 cm), washed with cyclohexane, and eluted with DCM. After removal of the volatiles under reduced pressure, the title compound was obtained as orange-red solid (117 mg, 15.2 µmol, 80%).

TLC: R_f = 0.48 in cyclohexane/DCM (1:1).

^{1}H NMR (400 MHz, $CDCl_3$): δ (ppm) = 7.78 (s, 2H, H_{ar}), 7.57–7.54 (m, 4H, H_{ar}), 7.39–7.32 (m, 6H, H_{ar}), 7.15 (s, 2H, H_{ar}), 7.06 (s, 2H, H_{ar}), 4.06 (t, J = 6.4 Hz, 4H, OCH_2CH_2), 4.04 (t, J = 6.4 Hz, 4H, OCH_2CH_2), 1.99–1.85 (m, 8H, $CH_2CH_2CH_3$), 1.14 (overlapped t, J = 7.6 Hz, 6H, CH_3), 1.12 (overlapped t, J = 7.7 Hz, 6H, CH_3).

^{13}C NMR (100 MHz, $CDCl_3$): δ (ppm) = 154.5 (C_q), 154.3 (C_q), 153.7 (C_q), 132.4 (*C*H), 131.8 (*C*H), 128.5 (*C*H), 123.5 (C_q), 117.4 (C_q), 117.3 (*C*H), 117.0 (*C*H), 115.2 (C_q), 113.3 (C_q), 95.5 (C_q), 94.5 (C_q), 91.0 (C_q), 86.0 (C_q), 71.4 (O*C*H_2), 71.3 (O*C*H_2), 22.9 (*C*H_2), 10.8 (*C*H_3), 10.7 (*C*H_3).

HRMS (ESI, *m*/*z*): $[M+H]^+$ calcd for $C_{50}H_{44}N_2O_4S$ 769.3095, found 769.3082.

IR (ATR): $\tilde{\nu}$ (cm^{-1}) = 2958 (w, C-H_{sp3}), 2933 (w, C-H_{sp3}), 2874 (w, C-H_{sp3}), 2203 (w, C≡C), 1510 (s, C=C_{ar}), 1485 (s), 1469 (m), 1415 (s), 1388 (s), 1323 (w), 1277 (s), 1216 (vs), 1065 (w), 1045 (m), 1024 (s), 1014 (s), 985 (w), 967 (m), 893 (m), 858 (s), 835 (s), 753 (vs), 689 (vs), 631 (m).

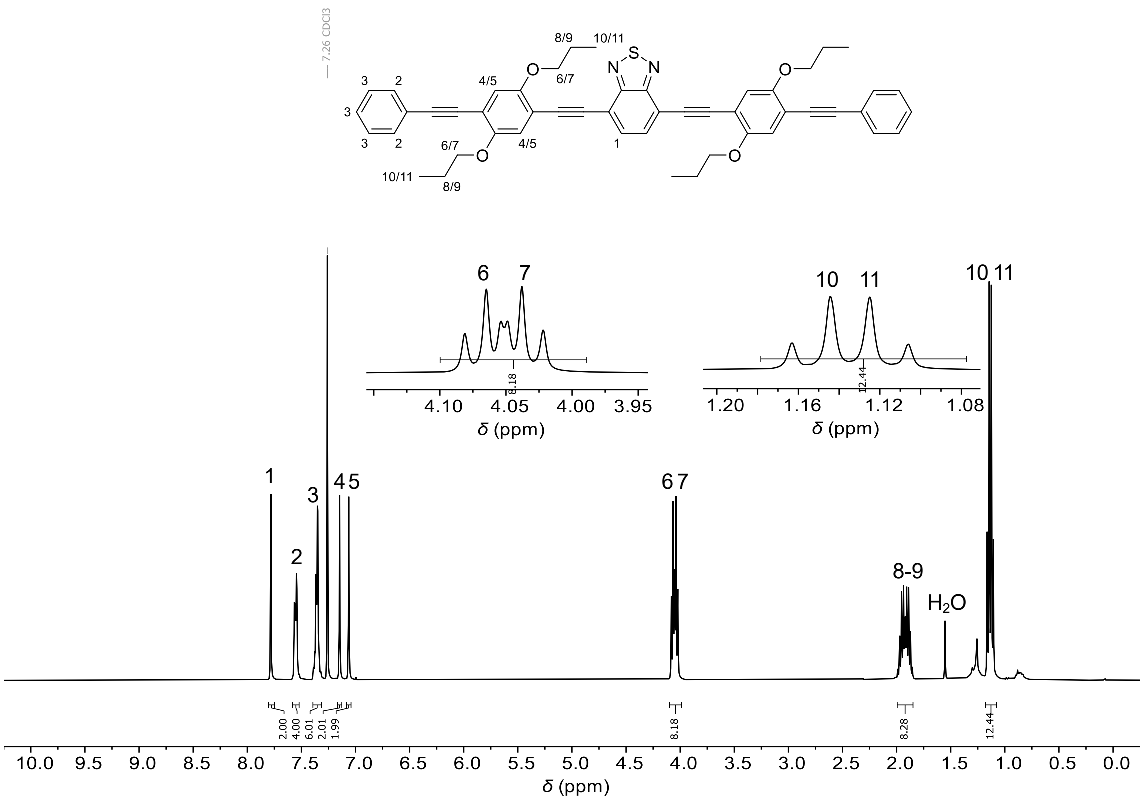

Figure S13. ^{1}H NMR spectrum of **OPE-y'**, measured in $CDCl_3$ at 400 MHz.

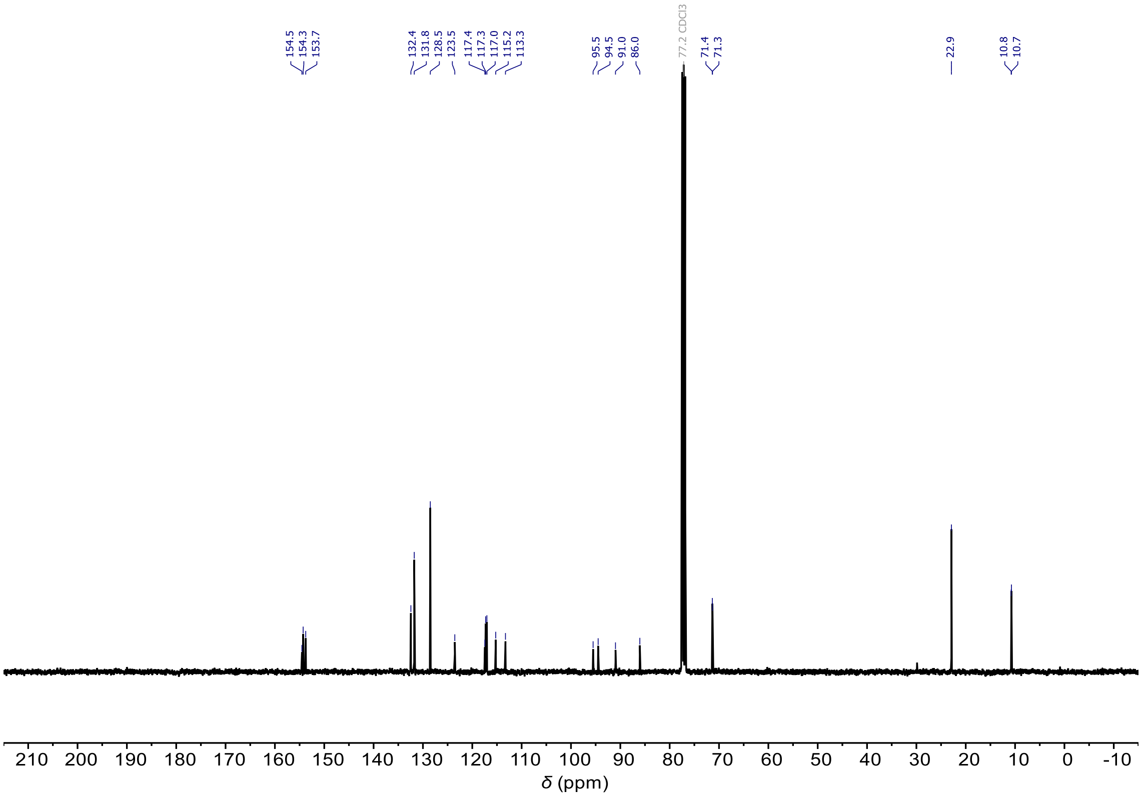

Figure S14. ^{13}C NMR spectrum of **OPE-y'**, measured in $CDCl_3$ at 101 MHz.

4,9-bis((4-(phenylethynyl)-2,5-dipropoxyphenyl)ethynyl)naphtho[2,3-*c*][1,2,5]thiadiazole (OPE-r')

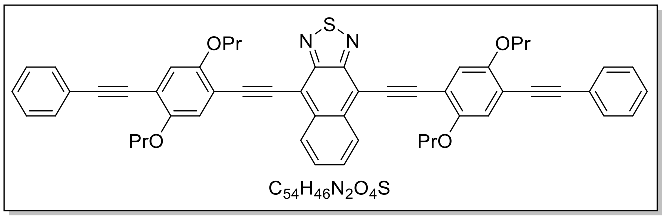

4,9-Dibromonaphtho[2,3-*c*][1,2,5]thiadiazole (60.5 mg, 176 µmol, 1.00 equiv.), **B1** (127 mg, 400 µmol, 2.28 equiv.), 5 mol% of $Pd(PPh_3)_4$ (9.9 mg, 8.6 µmol), and 10 mol% of copper(**I**) iodide (3.3 mg, 18 µmol) were degassed and suspended in 5 mL THF. Under continuous argon flow, diisopropylamine (0.50 mL, 0.36 g, 3.5 mmol, 20 equiv.) was added and the reaction mixture was stirred at room temperature for 20 h. The mixture was poured onto a short silica gel plug (5 cm) and washed with cyclohexane. Purification by flash column chromatography (cyclohexane → cyclohexane/DCM 1:3) yielded the title compound as a dark purple solid (78.2 mg, 138 µmol, 69%).

TLC (cyclohexane/DCM 5:1): R_f = 0.09.

^{1}H NMR (400 MHz, $CDCl_3$): δ = 7.67–7.53 (m, 4H, H_{ar}), 7.44–7.32 (m, 4H, H_{ar}), 7.32–7.18 (m, overlapped with solvent signal, H_{ar}), 7.10 (s, 2H, H_{ar}), 7.00 (s, 2H, H_{ar}), 4.14 (t, J = 6.5 Hz, 4H, OCH_2), 4.09 (t, J = 6.5 Hz, 4H, OCH_2), 2.13–1.99 (m, 4H, $CH_2CH_2CH_3$), 1.99–1.82 (m, 4H, $CH_2CH_2CH_3$), 1.19 (t, J = 7.4 Hz, 6H, CH_3), 1.15 (t, J = 7.4 Hz, 6H, CH_3).

^{13}C NMR: not available; spectra suffered from poor resolution under the conditions tested.

HRMS (ESI, *m/z*): $[M]^+$ calcd for $C_{54}H_{46}N_2O_2S$ 818.3178, found 818.3181.

IR (ATR): $\tilde{\nu}$ (cm^{-1}) = 2964 (w, C-H_{sp3}), 2909 (w, C-H_{sp3}), 2874 (w, C-H_{sp3}), 2184 (vw, C≡C), 1504 (m, C=C_{ar}), 1483 (w), 1471 (w), 1467 (w), 1444 (w), 1411 (m), 1390 (s), 1314 (w), 1275 (m), 1212 (vs), 1041 (m), 1022 (vs), 926 (w), 907 (w), 860 (s), 757 (vs), 691 (m), 650 (w), 529 (m).

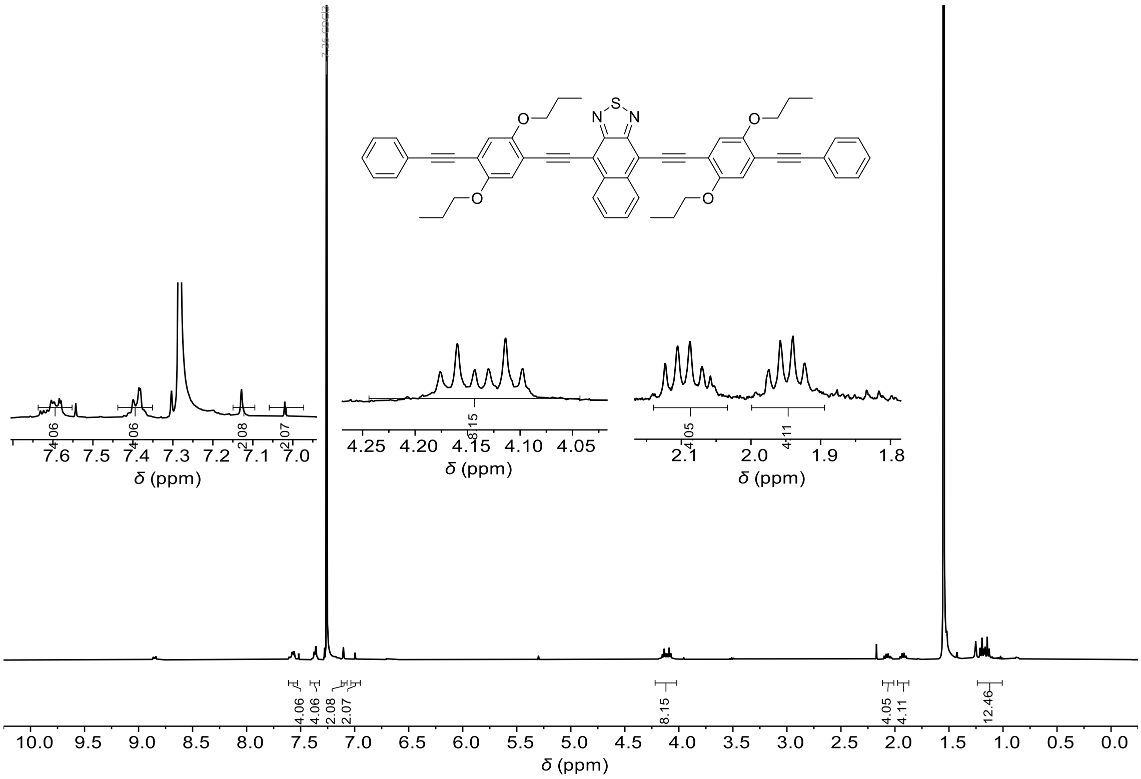

Figure S15. ^{1}H NMR spectrum of **OPE-r'**, measured in $CDCl_3$ at 400 MHz.

5 Quantum-chemical calculations

Calculations were submitted and processed using the Digichem software package,[1,2] which incorporates a number of publicly available software libraries, including: cclib[3] for the parsing of result files, VMD[4]/Tachyon[5] for 3D rendering, Matplotlib[6] for the plotting of graphs, Open Babel[7]/Pybel[8] for file interconversion, and PySOC[9] for the calculation of spin-orbit coupling.

Cartesian coordinates of optimized structures

OPE-b (S_0)

```
0       1
C          -1.41691000     0.06599000    -0.00001000
C          -0.73249000    -1.15973000    -0.00001000
C           0.64341000    -1.23916000    -0.00001000
C           1.41691000    -0.06600000    -0.00001000
C           0.73249000     1.15972000    -0.00001000
C          -0.64341000     1.23916000    -0.00001000
F           1.44664100     2.29024000    -0.00001000
F          -1.44664000    -2.29025000    -0.00001000
C          -2.82858000     0.11201000    -0.00001000
C           2.82858000    -0.11202000    -0.00001000
C          -4.04155000     0.17920100    -0.00001000
C           4.04155000    -0.17920000    -0.00001000
C          -5.45662000     0.22000100    -0.00001000
C           5.45662000    -0.22000000    -0.00001000
C          -6.13217000     1.45671100     0.00000000
C          -7.52744000     1.46868100     0.00000000
C          -8.24163000     0.27872100     0.00000000
C          -7.58110000    -0.95156900    -0.00001000
C          -6.19083000    -0.97712900    -0.00001000
C           6.13217000    -1.45671000     0.00000000
C           7.52744000    -1.46867000     0.00000000
C           8.24163000    -0.27872100     0.00000000
C           7.58109000     0.95157000    -0.00001000
C           6.19083000     0.97713000    -0.00001000
O          -5.35247900     2.56299100     0.00000000
C          -5.99599900     3.81480100     0.00005000
O          -8.37389000    -2.05466900    -0.00001000
C          -7.73492000    -3.30892900     0.00002000
O           8.37389000     2.05467900    -0.00001000
C           7.73491100     3.30894000     0.00002000
O           5.35248000    -2.56299000     0.00000000
C           5.99601000    -3.81480000     0.00005000
H           1.12765000    -2.20829000    -0.00001000
H          -1.12765000     2.20828000    -0.00001000
H          -8.07014900     2.40654100     0.00000000
H          -9.32651000     0.28383100     0.00000000
H          -5.63770000    -1.90805900    -0.00002000
H           8.07015000    -2.40653100     0.00000000
H           9.32651000    -0.28382100     0.00000000
H           5.63770000     1.90806000    -0.00002000
H          -5.20211900     4.56240100     0.00007000
H          -6.61817900     3.95078100    -0.89401000
H          -6.61816900     3.95072100     0.89411000
H          -8.52984000    -4.05564900     0.00004000
H          -7.11171000    -3.44670900    -0.89327000
H          -7.11170000    -3.44664900     0.89331000
H           8.52983100     4.05564900     0.00004000
H           7.11170100     3.44671000    -0.89327000
H           7.11170100     3.44666000     0.89331000
H           5.20212000    -4.56240000     0.00007000
H           6.61818000    -3.95078000    -0.89401000
H           6.61818000    -3.95071000     0.89411000
```

OPE-b (S_1)

0 1

C	-1.42783900	0.06889700	-0.00001400
C	-0.72801400	-1.16713800	-0.00001300
C	0.63575400	-1.25575800	-0.00000800
C	1.42783900	-0.06888300	-0.00001200
C	0.72801400	1.16715200	-0.00001300
C	-0.63575400	1.25577200	-0.00001100
F	1.45687200	2.29151200	-0.00001400
F	-1.45687100	-2.29149800	-0.00001300
C	-2.81743400	0.10919900	-0.00002200
C	2.81743400	-0.10918500	-0.00001800
C	-4.04439900	0.17506600	-0.00000100
C	4.04440000	-0.17505300	0.00000200
C	-5.43999900	0.20517400	-0.00000300
C	5.43999900	-0.20517100	-0.00000000
C	-6.13223400	1.45533500	0.00004100
C	-7.52623600	1.48161000	0.00005000
C	-8.24938500	0.29657700	0.00001300
C	-7.58401200	-0.93951100	-0.00003600
C	-6.19451400	-0.98603300	-0.00004500
C	6.13222500	-1.45533700	0.00004200
C	7.52622700	-1.48162200	0.00005000
C	8.24938400	-0.29659400	0.00001200
C	7.58402000	0.93949900	-0.00003500
C	6.19452300	0.98603000	-0.00004300
O	-5.34608800	2.54621100	0.00007600
C	-5.96392100	3.81342000	0.00013600
O	-8.38692400	-2.02866000	-0.00007100
C	-7.77027200	-3.29675600	-0.00010900
O	8.38694000	2.02864200	-0.00007100
C	7.77029700	3.29674300	-0.00011000
O	5.34607100	-2.54620800	0.00007600
C	5.96389600	-3.81342100	0.00013600
H	1.11561600	-2.22665200	-0.00000600
H	-1.11561600	2.22666600	-0.00001100
H	-8.05844600	2.42551200	0.00008700
H	-9.33386800	0.30178100	0.00002100
H	-5.65411900	-1.92379900	-0.00008000
H	8.05843000	-2.42552700	0.00008500
H	9.33386800	-0.30180500	0.00001800
H	5.65413400	1.92380000	-0.00007800
H	-5.15377900	4.54265900	0.00016700
H	-6.58272700	3.95623000	-0.89443000
H	-6.58272000	3.95614700	0.89472000
H	-8.57972400	-4.02725100	-0.00012400
H	-7.15014800	-3.44155900	-0.89348000
H	-7.15013800	-3.44160700	0.89324700
H	8.57975400	4.02723200	-0.00012700
H	7.15017300	3.44154900	-0.89348000
H	7.15016500	3.44159900	0.89324700
H	5.15374900	-4.54265400	0.00016700
H	6.58269900	-3.95623500	-0.89443200
H	6.58269500	-3.95615200	0.89471900

OPE-g (S_0)

0 1

C	-1.41905800	-0.03645900	-0.00039100
C	-0.68365500	-1.24698200	-0.00044800
C	0.74640500	-1.21181400	-0.00049000
C	1.41905700	0.03644400	-0.00050700
C	0.68365400	1.24696600	-0.00056000
C	-0.74640600	1.21179800	-0.00045400
C	1.33602900	2.50990200	-0.00074100

C	-1.33603000	-2.50991800	-0.00049600
C	-2.83043300	-0.05477200	-0.00026900
C	2.83043100	0.05475600	-0.00047300
C	-4.04662600	-0.00516500	-0.00012300
C	4.04662400	0.00515300	-0.00034300
C	-5.45597800	0.10605800	0.00013700
C	5.45597800	-0.10605800	0.00001500
C	-6.04964500	1.38545600	0.00021900
C	-7.43976100	1.49248500	0.00046700
C	-8.23288600	0.35303400	0.00063200
C	-7.65562000	-0.91826700	0.00055400
C	-6.27005700	-1.03804600	0.00030800
C	6.04965600	-1.38545100	0.00011400
C	7.43977300	-1.49246800	0.00045900
C	8.23288800	-0.35301100	0.00070800
C	7.65561100	0.91828600	0.00061500
C	6.27004700	1.03805300	0.00026800
C	-0.61685300	-3.67290100	-0.00054900
C	0.80008000	-3.63660800	-0.00057600
C	1.46362300	-2.44049900	-0.00053400
C	0.61685200	3.67288500	-0.00075500
C	-0.80008100	3.63659200	-0.00055800
C	-1.46362400	2.44048400	-0.00041800
O	-5.19674200	2.43893000	0.00005400
C	-5.75226900	3.73220700	-0.00000000
O	-8.52098500	-1.96576700	0.00072700
C	-7.96881000	-3.26011300	0.00068900
O	8.52096700	1.96579300	0.00087300
C	7.96878100	3.26013400	0.00084500
O	5.19676200	-2.43893300	-0.00015400
C	5.75230100	-3.73220400	0.00008700
H	2.42133400	2.52370500	-0.00088600
H	-2.42133500	-2.52372000	-0.00048400
H	-7.91783000	2.46495800	0.00053900
H	-9.31490800	0.43069300	0.00082600
H	-5.78375700	-2.00558600	0.00023800
H	7.91785000	-2.46493700	0.00053400
H	9.31491100	-0.43065900	0.00097800
H	5.78373900	2.00558800	0.00019300
H	-1.13098600	-4.62951100	-0.00059600
H	1.36048300	-4.56705000	-0.00061500
H	2.54905800	-2.40327800	-0.00055200
H	1.13098500	4.62949600	-0.00093300
H	-1.36048400	4.56703400	-0.00051700
H	-2.54905900	2.40326200	-0.00028200
H	-4.90837800	4.42299600	-0.00022900
H	-6.36318500	3.91012200	-0.89425000
H	-6.36287000	3.91033600	0.89442300
H	-8.81248700	-3.95130000	0.00087400
H	-7.35668100	-3.44013800	-0.89288000
H	-7.35635800	-3.44005200	0.89405400
H	8.81245100	3.95132900	0.00111300
H	7.35671200	3.44018700	-0.89276000
H	7.35626500	3.44003500	0.89417500
H	4.90841500	-4.42300100	-0.00007700
H	6.36328800	-3.91029200	-0.89408000
H	6.36283300	-3.91015100	0.89459300

OPE-g (S_1)

0 1

C	1.41822000	0.03152800	0.00002600
C	0.68777300	1.26150400	0.00003900
C	-0.74087800	1.23185400	0.00002300
C	-1.41822500	-0.03158800	-0.00001100
C	-0.68777800	-1.26156400	-0.00004300
C	0.74087300	-1.23191400	-0.00002000

C	-1.34022100	-2.51154500	-0.00009200
C	1.34021600	2.51148400	0.00006900
C	2.81342300	0.04448100	0.00005800
C	-2.81342800	-0.04454000	-0.00001500
C	4.03821200	-0.00219300	0.00003800
C	-4.03821600	0.00214500	-0.00002500
C	5.43477600	-0.10405600	0.00001400
C	-5.43477700	0.10405400	-0.00001300
C	6.04112500	-1.38933600	-0.00000400
C	7.43075200	-1.49550600	-0.00003200
C	8.22149900	-0.35450300	-0.00004000
C	7.63764500	0.91907500	-0.00002000
C	6.25482300	1.04269100	0.00000600
C	-6.04108300	1.38935500	-0.00004000
C	-7.43070600	1.49557100	-0.00002200
C	-8.22149100	0.35459400	0.00002700
C	-7.63768000	-0.91900300	0.00005700
C	-6.25486200	-1.04266600	0.00003300
C	0.62249200	3.69499800	0.00008800
C	-0.77806800	3.66427100	0.00007500
C	-1.44787300	2.45199500	0.00004500
C	-0.62249600	-3.69505900	-0.00011600
C	0.77806400	-3.66433100	-0.00008900
C	1.44786800	-2.45205500	-0.00004200
O	5.19201400	-2.43835000	0.00000900
C	5.74039100	-3.73627100	-0.00002700
O	8.50530700	1.96141700	-0.00003100
C	7.95922400	3.25961400	-0.00000400
O	-8.50537700	-1.96131600	0.00010700
C	-7.95933700	-3.25953100	0.00014300
O	-5.19193600	2.43834000	-0.00008800
C	-5.74027100	3.73627900	-0.00008100
H	-2.42576800	-2.52429700	-0.00010900
H	2.42576300	2.52423700	0.00007900
H	7.90898900	-2.46794100	-0.00004600
H	9.30379900	-0.42736200	-0.00006100
H	5.76959900	2.01052900	0.00002200
H	-7.90891100	2.46802200	-0.00004500
H	-9.30378900	0.42748900	0.00004700
H	-5.76967000	-2.01051900	0.00005400
H	1.14775600	4.64542100	0.00011000
H	-1.34256500	4.59215100	0.00009100
H	-2.53351200	2.42099900	0.00003500
H	-1.14776000	-4.64548100	-0.00015600
H	1.34256100	-4.59221000	-0.00010800
H	2.53350700	-2.42105900	-0.00002200
H	4.89176200	-4.42079000	-0.00002600
H	6.34982900	-3.91468700	-0.89475900
H	6.34986100	-3.91472400	0.89467500
H	8.80657200	3.94608400	-0.00001000
H	7.34781000	3.44040800	-0.89351600
H	7.34784200	3.44038400	0.89353400
H	-8.80670800	-3.94597300	0.00018400
H	-7.34796300	-3.44036600	-0.89338700
H	-7.34792700	-3.44030100	0.89366200
H	-4.89161900	4.42077000	-0.00009400
H	-6.34973100	3.91473400	-0.89479000
H	-6.34970600	3.91473500	0.89464400

OPE-y (S_0)

0 1

C	-1.46905400	0.33964900	-0.00020500
C	-0.76481100	1.53350700	-0.00039600
C	0.64386700	1.59064400	-0.00034300
C	1.43945800	0.45616200	-0.00009900
C	0.75542300	-0.80456000	0.00011600

C	-0.68652000	-0.86308200	0.00003900
C	-2.87701500	0.28933200	-0.00020100
C	2.84673800	0.51415700	-0.00007800
C	-4.09205800	0.24116900	-0.00019200
C	4.06145000	0.56320500	-0.00008100
C	-5.50560600	0.19217500	-0.00019800
C	5.47569900	0.56202200	-0.00005400
C	-6.16644700	-1.05391200	-0.00076400
C	-7.56182100	-1.08308100	-0.00071400
C	-8.29041100	0.09791800	-0.00010300
C	-7.64525100	1.33657700	0.00045500
C	-6.25577100	1.37997400	0.00039000
C	6.19420400	1.77443400	-0.00037800
C	7.58904800	1.73736600	-0.00034300
C	8.26077400	0.52286300	0.00000700
C	7.55744900	-0.68385200	0.00032900
C	6.16752000	-0.66074000	0.00029400
N	1.33682000	-2.00324900	0.00036500
S	0.12576300	-3.09229100	0.00051500
N	-1.16796500	-2.10501500	0.00028900
O	-5.37290500	-2.14598000	-0.00140700
C	-5.99158600	-3.40968200	-0.00098400
O	-8.45323800	2.42951700	0.00103500
C	-7.83144700	3.69157700	0.00161900
O	8.31115400	-1.81389400	0.00065200
C	7.62751700	-3.04467500	0.00101600
O	5.45398100	2.90752300	-0.00071300
C	6.14143700	4.13559500	-0.00093500
H	-1.32545800	2.46231200	-0.00066500
H	1.13053100	2.56017100	-0.00050100
H	-8.09186500	-2.02820000	-0.00118800
H	-9.37519900	0.07983500	-0.00006300
H	-5.71637700	2.31894600	0.00081900
H	8.16465900	2.65540000	-0.00059400
H	9.34519000	0.48985000	0.00003300
H	5.57971400	-1.57026400	0.00053900
H	-5.18131000	-4.13910800	-0.00086300
H	-6.61094200	-3.55739600	0.89329900
H	-6.61111900	-3.55793000	-0.89506100
H	-8.63600500	4.42796800	0.00203200
H	-7.20999200	3.83752700	0.89505600
H	-7.21010400	3.83840900	-0.89175200
H	8.39527800	-3.81929400	0.00125800
H	6.99959100	-3.15942800	0.89417300
H	6.99961200	-3.15996700	-0.89208700
H	5.37465300	4.91100400	-0.00110100
H	6.76812700	4.24962600	0.89307100
H	6.76816500	4.24928500	-0.89495800

OPE-y (S_1)

0	1		
C	-1.44251000	0.66431700	-0.00016800
C	-0.73024500	1.87908300	-0.00003800
C	0.65578200	1.90029900	0.00005300
C	1.39938100	0.70367700	0.00001400
C	0.70836500	-0.54790800	-0.00013400
C	-0.72329700	-0.56912800	-0.00021900
C	-2.84536600	0.64597400	-0.00026200
C	2.79974500	0.72210300	0.00015000
C	-4.06478700	0.57581000	-0.00029700
C	4.02068600	0.70196400	0.00027600
C	-5.44101600	0.30727300	-0.00007000
C	5.41703600	0.55507800	0.00012500
C	-5.84782700	-1.06344400	0.00009400
C	-7.21031900	-1.38402500	0.00032500
C	-8.15573000	-0.37575400	0.00039300

C	-7.76644900	0.97699500	0.00023300
C	-6.41649900	1.31316900	0.00000400
C	6.27232800	1.68657500	0.00008100
C	7.65620800	1.49978100	-0.00004900
C	8.18808300	0.21991600	-0.00013400
C	7.35203300	-0.90661200	-0.00008900
C	5.97422400	-0.73658500	0.00003900
N	1.29326300	-1.75407900	-0.00017200
S	0.02976800	-2.84170800	-0.00034300
N	-1.26990800	-1.79528400	-0.00036100
O	-4.85168500	-1.94409500	0.00001800
C	-5.13503700	-3.32698700	0.00021400
O	-8.78039100	1.86684800	0.00032100
C	-8.45219800	3.23988100	0.00016600
O	7.98163100	-2.10442300	-0.00017200
C	7.17325800	-3.26110200	-0.00012400
O	5.66081900	2.88589900	0.00017500
C	6.46862500	4.04107300	0.00014800
H	-1.28919200	2.80878200	0.00000200
H	1.19001800	2.84386100	0.00016400
H	-7.53028900	-2.41930800	0.00045300
H	-9.21629700	-0.60333900	0.00057300
H	-6.08601000	2.34386700	-0.00012100
H	8.32790900	2.34988400	-0.00008400
H	9.26183500	0.06486800	-0.00023300
H	5.28785800	-1.57414200	0.00008400
H	-4.16211000	-3.81597100	0.00013700
H	-5.69715500	-3.61596400	0.89620700
H	-5.69739800	-3.61616900	-0.89556000
H	-9.40069400	3.77677800	0.00027000
H	-7.87900700	3.51359400	0.89425500
H	-7.87927900	3.51345800	-0.89413800
H	7.85854200	-4.10908300	-0.00020400
H	6.53796900	-3.30734500	0.89298600
H	6.53780800	-3.30730600	-0.89312100
H	5.78183400	4.88755400	0.00024500
H	7.10209600	4.08820400	0.89468900
H	7.10193300	4.08827700	-0.89450600

OPE-r (S_0)

0 1

C	1.45411700	-0.01908600	-0.00001000
C	0.72389100	1.19237600	-0.00002200
C	-0.71904400	1.19887600	0.00005300
C	-1.45749000	-0.00749600	0.00011200
C	-0.73014200	-1.22428600	0.00009500
C	0.71467400	-1.23064700	0.00005400
C	1.40556300	2.44395300	-0.00004500
C	2.85856900	-0.05073600	-0.00005500
C	-2.86152400	-0.00720400	0.00018500
C	4.07562500	-0.08792500	-0.00007500
C	-4.07672000	0.05695800	0.00020000
C	5.48737000	-0.13943700	-0.00005600
C	-5.48538500	0.15087400	0.00007100
C	6.14358500	-1.38885500	-0.00007200
C	7.53864700	-1.42411900	-0.00005600
C	8.27237200	-0.24624800	-0.00002300
C	7.63241800	0.99542400	-0.00000600
C	6.24346300	1.04524600	-0.00002300
C	-6.10000800	1.42037700	-0.00001300
C	-7.49196600	1.50265700	-0.00013200
C	-8.26369800	0.34870800	-0.00016900
C	-7.66460600	-0.91323200	-0.00008600
C	-6.27767600	-1.00902200	0.00003300
C	0.72045700	3.62390600	-0.00004400
C	-0.70033000	3.62849300	0.00003200

C	-1.39587300	2.45436100	0.00007000
N	-1.26761200	-2.45227700	0.00014500
S	-0.01787200	-3.48550200	0.00009900
N	1.23953300	-2.46379900	0.00005600
O	5.34486300	-2.47652900	-0.00010500
C	5.95632200	-3.74375200	-0.00011500
O	8.44541100	2.08454600	0.00002500
C	7.82952000	3.34947000	0.00004800
O	-8.51174900	-1.97459000	-0.00012900
C	-7.93634800	-3.25963800	-0.00004400
O	-5.26534600	2.48807400	0.00003100
C	-5.84438700	3.77125000	-0.00003300
H	2.49059200	2.42729400	-0.00006300
H	8.06442200	-2.37162400	-0.00006900
H	9.35705200	-0.26883800	-0.00001100
H	5.70941800	1.98734300	-0.00000900
H	-7.98797800	2.46606400	-0.00019900
H	-9.34698000	0.40706700	-0.00026200
H	-5.77032200	-1.96563200	0.00010100
H	1.26120500	4.56557200	-0.00007000
H	-1.23366900	4.57444700	0.00003400
H	-2.48196700	2.44646800	0.00012600
H	5.14165300	-4.46822100	-0.00013700
H	6.57486000	-3.89515000	-0.89432500
H	6.57483500	-3.89517600	0.89410800
H	8.63748100	4.08210300	0.00007400
H	7.20889100	3.49874000	-0.89344000
H	7.20887100	3.49869800	0.89352900
H	-8.76786900	-3.96532000	-0.00009400
H	-7.32074700	-3.42814600	-0.89322800
H	-7.32089700	-3.42809500	0.89325300
H	-5.01343500	4.47748400	0.00002900
H	-6.45828800	3.93798600	-0.89435900
H	-6.45843800	3.93800700	0.89418600

OPE-r (S_1)

0 1

C	-1.43989200	0.14943300	0.00000200
C	-0.72107500	1.38239000	-0.00003800
C	0.71373600	1.37904600	-0.00002400
C	1.42376000	0.14023900	0.00002700
C	0.70552600	-1.08514200	0.00006100
C	-0.72974200	-1.08135300	0.00004900
C	-1.39374500	2.62476200	-0.00008600
C	-2.83800000	0.12181100	-0.00000400
C	2.82031500	0.12306300	0.00003600
C	-4.05841300	0.06202100	-0.00002300
C	4.04153300	0.13846200	-0.00000600
C	-5.45104300	-0.10803500	-0.00001900
C	5.44437600	0.13491700	0.00000000
C	-5.97492700	-1.43107500	0.00001800
C	-7.35945100	-1.62242200	0.00001900
C	-8.21199700	-0.53140700	-0.00001800
C	-7.70640600	0.77820800	-0.00005600
C	-6.33288400	0.98586800	-0.00005400
C	6.15583300	1.36174100	0.00005800
C	7.55126300	1.34354200	0.00006000
C	8.23352200	0.13657400	0.00000400
C	7.54096400	-1.08255900	-0.00005500
C	6.15264800	-1.08059200	-0.00005400
C	-0.69718200	3.81404900	-0.00011900
C	0.70973700	3.80917200	-0.00010300
C	1.39881100	2.61530500	-0.00005700
N	1.26198100	-2.30440300	0.00010900
S	-0.01573300	-3.36162200	0.00013900
N	-1.28964900	-2.30001100	0.00009200

O	-5.06332700	-2.40918800	0.00004800
C	-5.49906500	-3.75015200	0.00007600
O	-8.63626900	1.76160600	-0.00009100
C	-8.17577100	3.09389700	-0.00012600
O	8.31170300	-2.19544700	-0.00011000
C	7.65075000	-3.44155500	-0.00017400
O	5.40332000	2.48007700	0.00010900
C	6.06538200	3.72499300	0.00016900
H	-2.47932500	2.61861600	-0.00009800
H	-7.77564100	-2.62292500	0.00004600
H	-9.28849600	-0.66613300	-0.00001900
H	-5.90726500	1.98134200	-0.00008300
H	8.11581400	2.26832900	0.00010400
H	9.31815900	0.11264100	0.00000300
H	5.57543000	-1.99660300	-0.00009800
H	-1.23663600	4.75643800	-0.00015800
H	1.25494700	4.74821900	-0.00012700
H	2.48494100	2.60096600	-0.00004300
H	-4.59032800	-4.35095200	0.00009400
H	-6.09061200	-3.97800200	0.89536500
H	-6.09060500	-3.97804300	-0.89520600
H	-9.06644200	3.72264200	-0.00014900
H	-7.57804300	3.31266400	0.89377000
H	-7.57803200	3.31261300	-0.89402700
H	8.43411300	-4.19987100	-0.00021100
H	7.02599000	-3.56560800	0.89318000
H	7.02599200	-3.56551700	-0.89354100
H	5.28092800	4.48185300	0.00020200
H	6.68785600	3.84825600	0.89499900
H	6.68786100	3.84833900	-0.89464500

OPE-b’ (S_0)

0 1

C	-1.41768400	-0.03436300	0.00000700
C	-0.70612600	1.17636000	0.00000600
C	0.67086700	1.22512100	0.00000600
C	1.41768400	0.03436500	0.00000600
C	0.70612600	-1.17635800	0.00000700
C	-0.67086700	-1.22511900	0.00000700
C	-2.82906400	-0.04847900	0.00000700
C	2.82906400	0.04848200	0.00000600
C	-4.04385600	-0.08829100	0.00000700
C	4.04385600	0.08829400	0.00000600
C	-5.45667300	-0.09845900	0.00000600
C	5.45667300	0.09846000	0.00000600
C	-6.16277700	-1.32079600	0.00000400
C	-7.55096800	-1.30458900	0.00000300
C	-8.26352500	-0.09576100	0.00000400
C	-7.55612900	1.12704000	0.00000700
C	-6.16857400	1.11155000	0.00000900
C	6.16277800	1.32079700	0.00000200
C	7.55096900	1.30459000	0.00000200
C	8.26352500	0.09576100	0.00000400
C	7.55612900	-1.12703900	0.00000700
C	6.16857400	-1.11154800	0.00000900
F	1.39541300	-2.32181100	0.00000700
F	-1.39541300	2.32181400	0.00000600
C	-9.67782000	-0.10158400	0.00000300
C	9.67782000	0.10158400	0.00000200
C	-10.89309900	-0.12102900	0.00000100
C	10.89310000	0.12102800	0.00000100
C	-12.31244900	-0.11748700	-0.00000100
C	12.31244900	0.11748400	-0.00000100
C	-13.03362000	-1.32279900	-0.00000600
C	-14.42146400	-1.30751100	-0.00000800
C	-15.11093600	-0.09689000	-0.00000400

C	-14.40356100	1.10357800	0.00000200
C	-13.01584100	1.09878000	0.00000300
C	13.03362100	1.32279500	-0.00000800
C	14.42146500	1.30750600	-0.00001000
C	15.11093600	0.09688400	-0.00000500
C	14.40356000	-1.10358300	0.00000200
C	13.01584000	-1.09878300	0.00000400
O	-5.40799300	-2.44297000	0.00000400
C	-6.08016000	-3.68062000	-0.00003200
O	-8.31036300	2.24936400	0.00001000
C	-7.63654600	3.48621300	-0.00001600
O	8.31036200	-2.24936400	0.00001100
C	7.63654300	-3.48621200	-0.00001700
O	5.40799400	2.44297100	0.00000200
C	6.08016200	3.68062200	-0.00003100
H	1.17682100	2.18307100	0.00000600
H	-1.17682100	-2.18306800	0.00000800
H	-8.12097700	-2.22498200	0.00000300
H	-5.59708100	2.03108400	0.00001300
H	8.12097800	2.22498300	0.00000100
H	5.59708000	-2.03108200	0.00001400
H	-12.49078100	-2.26241500	-0.00000900
H	-14.96919500	-2.24520300	-0.00001200
H	-16.19672100	-0.08898000	-0.00000500
H	-14.93749800	2.04919800	0.00000500
H	-12.45625300	2.02838000	0.00000800
H	12.49078300	2.26241200	-0.00001200
H	14.96919700	2.24519700	-0.00001500
H	16.19672100	0.08897400	-0.00000600
H	14.93749600	-2.04920300	0.00000600
H	12.45625100	-2.02838300	0.00000900
H	-5.30323700	-4.44566700	-0.00005100
H	-6.70517000	-3.80100100	0.89405600
H	-6.70516700	-3.80095100	-0.89412800
H	-8.41258300	4.25215400	-0.00003200
H	-7.01117000	3.60592200	0.89391000
H	-7.01117000	3.60588400	-0.89394700
H	8.41257900	-4.25215400	-0.00003400
H	7.01116700	-3.60592200	0.89390900
H	7.01116700	-3.60588100	-0.89394800
H	5.30323900	4.44566800	-0.00004900
H	6.70517200	3.80100000	0.89405700
H	6.70516900	3.80095300	-0.89412800

OPE-b' (S_1)

0 1

C	1.42665100	-0.04026500	-0.00025300
C	0.70396200	1.18163400	-0.00023900
C	-0.66189400	1.23994300	-0.00023800
C	-1.42665100	0.04030900	-0.00024700
C	-0.70396100	-1.18159000	-0.00023900
C	0.66189400	-1.23989900	-0.00024500
C	2.81545500	-0.05209800	-0.00030900
C	-2.81545500	0.05214100	-0.00029700
C	4.04317900	-0.09086300	-0.00036800
C	-4.04317900	0.09090200	-0.00035300
C	5.43405900	-0.09290300	-0.00022200
C	-5.43405900	0.09292700	-0.00020700
C	6.15560300	-1.32697900	-0.00015600
C	7.53720400	-1.32077500	-0.00003800
C	8.26028700	-0.10965000	0.00002200
C	7.54324800	1.12180100	-0.00003800
C	6.16122500	1.11949600	-0.00015200
C	-6.15561700	1.32699500	-0.00014100
C	-7.53721700	1.32077500	-0.00002400
C	-8.26028700	0.10964200	0.00003600

C	-7.54323400	-1.12180000	-0.00002400
C	-6.16121200	-1.11948000	-0.00013700
F	-1.40820200	-2.31930900	-0.00024000
F	1.40820200	2.31935300	-0.00023900
C	9.66085900	-0.11120000	0.00013200
C	-9.66086000	0.11117700	0.00014500
C	10.88242300	-0.12731600	0.00021900
C	-10.88242300	0.12728000	0.00022800
C	12.29249600	-0.11912900	0.00029400
C	-12.29249600	0.11908800	0.00029400
C	13.02234800	-1.32473400	0.00033100
C	14.40821900	-1.30303400	0.00040300
C	15.09478000	-0.08889900	0.00044000
C	14.38214000	1.11030200	0.00040500
C	12.99630400	1.10238700	0.00033300
C	-13.02235300	1.32469000	0.00032700
C	-14.40822400	1.30298400	0.00038900
C	-15.09478100	0.08884600	0.00042200
C	-14.38213500	-1.11035200	0.00039100
C	-12.99630000	-1.10243100	0.00032800
O	5.39204400	-2.43690600	-0.00022200
C	6.04741400	-3.68482900	-0.00016300
O	8.30651700	2.23299800	0.00002800
C	7.64763500	3.47960700	-0.00002300
O	-8.30649100	-2.23300600	0.00004100
C	-7.64759500	-3.47960800	-0.00001000
O	-5.39207000	2.43693000	-0.00020700
C	-6.04745400	3.68484600	-0.00014900
H	-1.16302000	2.20016400	-0.00024300
H	1.16302000	-2.20012000	-0.00025300
H	8.10184800	-2.24433500	0.00000900
H	5.59584100	2.04234100	-0.00020200
H	-8.10187200	2.24433000	0.00002200
H	-5.59581700	-2.04231900	-0.00018800
H	12.48317300	-2.26644200	0.00030200
H	14.95983400	-2.23854300	0.00043100
H	16.18046800	-0.07731300	0.00049600
H	14.91353400	2.05745100	0.00043300
H	12.43400400	2.03035100	0.00030400
H	-12.48318200	2.26640000	0.00030000
H	-14.95984300	2.23849100	0.00041300
H	-16.18046800	0.07725600	0.00047000
H	-14.91352500	-2.05750300	0.00041600
H	-12.43399500	-2.03039400	0.00030400
H	5.25993600	-4.43862200	-0.00022700
H	6.67081000	-3.80948200	-0.89435800
H	6.67066000	-3.80947600	0.89413600
H	8.43347700	4.23517500	0.00004800
H	7.02399500	3.60335000	-0.89399400
H	7.02384100	3.60334200	0.89384200
H	-8.43342800	-4.23518500	0.00006100
H	-7.02395300	-3.60334400	-0.89398100
H	-7.02380000	-3.60333600	0.89385600
H	-5.25998500	4.43864700	-0.00021200
H	-6.67085100	3.80949200	-0.89434400
H	-6.67070200	3.80948600	0.89415000

OPE-g' (S_0)

0 1

C	-15.09821900	0.27916900	0.00038100
C	-14.44306300	-0.95058700	0.00037400
C	-13.05644900	-1.00552700	0.00030700
C	-12.30113400	0.17928100	0.00024600
C	-12.96995200	1.41448400	0.00025300
C	-14.35716500	1.45891900	0.00032000
C	-10.88309700	0.12142600	0.00017900

C	-9.66968800	0.04901800	0.00012300
C	-8.25741100	-0.01905800	0.00005900
C	-7.60379200	-1.27144000	0.00005300
C	-6.21688300	-1.31827900	-0.00000700
C	-5.45084800	-0.14178600	-0.00006400
C	-6.10512100	1.11072200	-0.00005600
C	-7.49136900	1.15733900	0.00000500
C	-4.04119600	-0.19021400	-0.00012900
C	-2.82320900	-0.18448700	-0.00018000
C	-1.41567400	-0.10171700	-0.00022400
C	-0.62542000	-1.27800900	-0.00025300
C	0.80114800	-1.17720700	-0.00024500
C	1.41566400	0.10148100	-0.00023300
C	0.62541100	1.27777000	-0.00025900
C	-0.80116100	1.17696700	-0.00023400
C	-1.21916300	-2.56916400	-0.00030100
C	-0.44723600	-3.69803600	-0.00033000
C	0.96624500	-3.59669900	-0.00030300
C	1.57413600	-2.37118800	-0.00025900
C	15.09823400	-0.27882600	0.00037500
C	14.44303900	0.95090900	0.00038200
C	13.05642400	1.00580600	0.00031500
C	12.30114500	-0.17902600	0.00023900
C	12.97000200	-1.41420800	0.00023300
C	14.35721700	-1.45859900	0.00030100
C	10.88310600	-0.12121200	0.00017100
C	9.66969500	-0.04885400	0.00011500
C	8.25741400	0.01913200	0.00005000
C	7.60371400	1.27146900	0.00005400
C	6.21680100	1.31821800	-0.00001100
C	5.45084000	0.14168000	-0.00007700
C	6.10519700	-1.11078800	-0.00008000
C	7.49144500	-1.15731600	-0.00001900
C	4.04118500	0.19002500	-0.00013900
C	2.82319900	0.18424700	-0.00018900
C	1.21915300	2.56892900	-0.00032000
C	0.44722800	3.69780000	-0.00032800
C	-0.96625500	3.59646400	-0.00026500
C	-1.57414700	2.37095500	-0.00021900
O	-5.30088900	2.20055700	-0.00010500
C	-5.91553100	3.46778800	-0.00018600
O	-8.40731300	-2.35931000	0.00010800
C	-7.79026900	-3.62493600	0.00012700
O	5.30103700	-2.20067400	-0.00015200
C	5.91577500	-3.46785800	-0.00005200
O	8.40716100	2.35939500	0.00012000
C	7.79003100	3.62497900	0.00015900
H	-16.18333700	0.31801400	0.00043300
H	-15.01716400	-1.87239100	0.00042100
H	-12.53745400	-1.95839200	0.00030200
H	-12.38725900	2.32992600	0.00020600
H	-14.86396700	2.41936400	0.00032500
H	-5.68959100	-2.26383800	-0.00001400
H	-8.01978200	2.10230400	0.00001600
H	-2.30264500	-2.63315400	-0.00032200
H	-0.91690600	-4.67720000	-0.00037500
H	1.56884700	-4.50032600	-0.00032300
H	2.65671800	-2.28450400	-0.00024300
H	16.18335200	-0.31763800	0.00042800
H	15.01711100	1.87273100	0.00044000
H	12.53739800	1.95865500	0.00031900
H	12.38733800	-2.32966800	0.00017500
H	14.86404800	-2.41902800	0.00029500
H	5.68945500	2.26374700	-0.00000800
H	8.01991700	-2.10224800	-0.00002400
H	2.30263600	2.63292000	-0.00036600
H	0.91689900	4.67696300	-0.00039200
H	-1.56885600	4.50009200	-0.00024800
H	-2.65672900	2.28427400	-0.00017300

H	-5.10401800	4.19618100	-0.00028000
H	-6.53381800	3.61610400	-0.89453400
H	-6.53374300	3.61625600	0.89419000
H	-8.59999600	-4.35520600	0.00019000
H	-7.17109600	-3.77302000	-0.89400500
H	-7.17101600	-3.77295300	0.89421400
H	5.10431800	-4.19631400	-0.00005200
H	6.53408000	-3.61625200	-0.89437500
H	6.53399300	-3.61615100	0.89434800
H	8.59970900	4.35530300	0.00023400
H	7.17084800	3.77303500	-0.89397100
H	7.17076700	3.77294000	0.89424800

OPE-g' (S_1)

0	1		
C	15.08082000	-0.26818400	-0.00001600
C	14.42082200	0.96015800	0.00000500
C	13.03529600	1.01145500	-0.00003600
C	12.28090900	-0.17761900	-0.00009900
C	12.95676600	-1.41251000	-0.00012000
C	14.34292200	-1.45114300	-0.00007800
C	10.86893400	-0.12463700	-0.00014300
C	9.65120000	-0.05552500	-0.00017600
C	8.24829600	0.00778200	-0.00016700
C	7.58722100	1.26588700	-0.00011800
C	6.20551900	1.31908500	-0.00010600
C	5.42979500	0.13906400	-0.00013500
C	6.09555900	-1.12121500	-0.00019500
C	7.47717300	-1.17118200	-0.00020600
C	4.03660300	0.18174500	-0.00010000
C	2.80914300	0.17560600	-0.00004600
C	1.41992200	0.09813100	0.00004400
C	0.62711800	1.28989400	0.00016600
C	-0.79672600	1.19349500	0.00027000
C	-1.41991600	-0.09801900	0.00025100
C	-0.62711100	-1.28977600	0.00012600
C	0.79673500	-1.19337600	0.00002300
C	1.22118000	2.57020600	0.00017900
C	0.45104300	3.71464100	0.00030000
C	-0.95020800	3.61815800	0.00041000
C	-1.56117600	2.38062200	0.00039400
C	-15.08083000	0.26807900	-0.00042800
C	-14.42082900	-0.96026200	-0.00044500
C	-13.03530400	-1.01155500	-0.00033500
C	-12.28091900	0.17752200	-0.00020800
C	-12.95677900	1.41241000	-0.00019200
C	-14.34293500	1.45103900	-0.00030100
C	-10.86894400	0.12454000	-0.00009800
C	-9.65121000	0.05543200	-0.00001100
C	-8.24830400	-0.00783400	0.00009300
C	-7.58719200	-1.26591800	0.00007000
C	-6.20548600	-1.31907800	0.00015400
C	-5.42979400	-0.13904400	0.00026300
C	-6.09559900	1.12122100	0.00029600
C	-7.47721300	1.17115300	0.00020900
C	-4.03660100	-0.18167200	0.00032400
C	-2.80913800	-0.17550500	0.00032400
C	-1.22116700	-2.57009500	0.00011000
C	-0.45102900	-3.71452700	-0.00001700
C	0.95022400	-3.61804400	-0.00013200
C	1.56118900	-2.38050700	-0.00011200
O	5.28865200	-2.20421100	-0.00023000
C	5.89433200	-3.47666900	-0.00030200
O	8.39599600	2.34695200	-0.00008800
C	7.78707100	3.61743700	-0.00003000
O	-5.28871900	2.20423400	0.00040700

C	-5.89441900	3.47668100	0.00045700
O	-8.39593100	-2.34700900	-0.00004100
C	-7.78696900	-3.61747700	-0.00007100
H	16.16602000	-0.30340400	0.00001600
H	14.99227700	1.88367100	0.00005300
H	12.51340100	1.96272900	-0.00002000
H	12.37744100	-2.33007300	-0.00016900
H	14.85341300	-2.40969500	-0.00009500
H	5.68066500	2.26577200	-0.00006700
H	8.00380900	-2.11709300	-0.00024400
H	2.30499700	2.63291800	0.00009300
H	0.92973100	4.68932000	0.00030800
H	-1.55603200	4.51955000	0.00050700
H	-2.64424200	2.29846200	0.00047600
H	-16.16603000	0.30329600	-0.00051400
H	-14.99228200	-1.88377600	-0.00054300
H	-12.51340500	-1.96282700	-0.00034700
H	-12.37745700	2.32997500	-0.00009200
H	-14.85342800	2.40959000	-0.00028800
H	-5.68061400	-2.26575500	0.00013300
H	-8.00387700	2.11704700	0.00022300
H	-2.30498400	-2.63280700	0.00019800
H	-0.92971500	-4.68920800	-0.00002800
H	1.55605000	-4.51943300	-0.00023700
H	2.64425600	-2.29834500	-0.00019800
H	5.07737500	-4.19871800	-0.00033500
H	6.51154700	-3.62672800	-0.89482100
H	6.51155700	-3.62682400	0.89419400
H	8.60164900	4.34212600	-0.00001000
H	7.16870800	3.76730600	-0.89410600
H	7.16872700	3.76723400	0.89407100
H	-5.07747300	4.19874300	0.00056100
H	-6.51158200	3.62684200	-0.89408100
H	-6.51169900	3.62671500	0.89493600
H	-8.60152700	-4.34218900	-0.00016000
H	-7.16855600	-3.76724400	-0.89412900
H	-7.16866700	-3.76734000	0.89404800

OPE-y' (S_0)

0 1

C	1.46001000	0.29423400	0.00007300
C	0.73682800	1.47767200	0.00017000
C	-0.67168400	1.51239700	0.00013800
C	-1.44922300	0.36448500	0.00001400
C	-0.74553000	-0.88553800	-0.00009800
C	0.69693700	-0.92112100	-0.00007300
C	2.86746600	0.26678000	0.00010800
C	-2.85608500	0.40077300	-0.00000300
C	4.08382500	0.23873500	0.00013600
C	-4.07205900	0.43218900	-0.00000500
C	5.49500600	0.21265700	0.00015100
C	-5.48317400	0.41242800	-0.00000100
C	6.18042000	-1.02298100	0.00039100
C	7.56895400	-1.03094100	0.00036800
C	8.30231600	0.16529600	0.00009800
C	7.61641300	1.40060200	-0.00013400
C	6.22920700	1.40992400	-0.00009400
C	-6.22143500	1.61595400	0.00011000
C	-7.60857800	1.56306200	0.00011300
C	-8.28875100	0.33551900	0.00000400
C	-7.54923300	-0.86858100	-0.00010900
C	-6.16297200	-0.81658000	-0.00010800
C	9.71614400	0.13557600	0.00007200
C	-9.70248600	0.30406600	0.00001400
C	10.93099900	0.09600100	0.00005300
C	-10.91794100	0.29174500	0.00002500

C	12.35011600	0.07593700	0.00002700
C	-12.33660800	0.25116100	0.00003700
C	13.05127600	-1.14115500	0.00026500
C	14.43917600	-1.14878000	0.00023600
C	15.14858600	0.05027000	-0.00002900
C	14.46116300	1.26228700	-0.00026600
C	13.07358000	1.28041300	-0.00023900
C	-13.08900100	1.43727300	0.00016700
C	-14.47596200	1.38584200	0.00017900
C	-15.13368100	0.15767900	0.00006200
C	-14.39524900	-1.02395100	-0.00006700
C	-13.00814100	-0.98303100	-0.00008000
N	-1.30778700	-2.09315900	-0.00023700
S	-0.07963500	-3.16297600	-0.00033600
N	1.19806700	-2.15506500	-0.00022100
O	5.40577000	-2.12735400	0.00068800
C	6.04713500	-3.38063100	0.00023300
O	8.39163900	2.50940400	-0.00038600
C	7.74113900	3.75786000	-0.00062400
O	-8.27373500	-2.01019100	-0.00020900
C	-7.56662200	-3.22862400	-0.00035600
O	-5.49666200	2.75774300	0.00021200
C	-6.20167100	3.97680900	0.00024500
H	1.28292000	2.41507900	0.00030600
H	-1.17403900	2.47386200	0.00022000
H	8.12179400	-1.96181100	0.00058300
H	5.67599900	2.34056600	-0.00027600
H	-8.20305000	2.46786000	0.00020400
H	-5.56504800	-1.71925200	-0.00019000
H	12.49295300	-2.07164700	0.00047100
H	14.97131000	-2.09540900	0.00042100
H	16.23435500	0.04021200	-0.00005000
H	15.01068700	2.19894300	-0.00047200
H	12.52940200	2.21912300	-0.00042200
H	-12.57080800	2.39070700	0.00025800
H	-15.04792600	2.30896000	0.00027900
H	-16.21889300	0.12147400	0.00007200
H	-14.90436400	-1.98316700	-0.00015800
H	-12.42451100	-1.89772900	-0.00017900
H	5.24974200	-4.12396000	0.00002800
H	6.66901100	-3.51587300	-0.89404800
H	6.66910100	-3.51649200	0.89436200
H	8.53091700	4.50969800	-0.00079800
H	7.11798000	3.88923900	-0.89467500
H	7.11802900	3.88960300	0.89340800
H	-8.32180000	-4.01514600	-0.00044400
H	-6.93808800	-3.33096700	-0.89413500
H	-6.93807800	-3.33117800	0.89339400
H	-5.44559100	4.76249700	0.00026300
H	-6.82978600	4.08046900	-0.89379200
H	-6.82978000	4.08042600	0.89429200

OPE-y' (S_1)

0 1

C	-1.43613000	0.64006700	-0.00033300
C	-0.72678800	1.85897800	-0.00024400
C	0.65822300	1.89408600	-0.00015300
C	1.42382300	0.71026100	-0.00014900
C	0.74245900	-0.54707500	-0.00024300
C	-0.69589000	-0.58361900	-0.00033500
C	-2.83502300	0.61776600	-0.00041200
C	2.82155000	0.74472800	-0.00005500
C	-4.05729000	0.57794600	-0.00044900
C	4.04415200	0.74014000	0.00000800
C	-5.45015000	0.45486100	-0.00033100
C	5.43732100	0.61470700	0.00004300

C	-6.03057100	-0.85044600	-0.00026700
C	-7.40879500	-0.99184400	-0.00013500
C	-8.24997100	0.13372800	-0.00006200
C	-7.67302900	1.43433600	-0.00013500
C	-6.29480100	1.57826000	-0.00026800
C	6.28088300	1.76199500	0.00010200
C	7.65642600	1.60290300	0.00013700
C	8.23737500	0.32235100	0.00011600
C	7.39609500	-0.82395900	0.00005900
C	6.02031600	-0.66582000	0.00002200
C	-9.64737000	-0.01615300	0.00006900
C	9.63492100	0.17152600	0.00015500
C	-10.85782500	-0.15915400	0.00019300
C	10.84780300	0.05162400	0.00019200
C	-12.26456300	-0.30224400	0.00035100
C	12.25206300	-0.11552700	0.00019900
C	-12.85683700	-1.57834400	0.00044000
C	-14.23768300	-1.70645100	0.00059500
C	-15.04893800	-0.57277900	0.00066400
C	-14.47108800	0.69601800	0.00057600
C	-13.09159700	0.83633800	0.00042100
C	13.10953200	0.99968600	0.00025000
C	14.48499200	0.82239700	0.00025800
C	15.02843800	-0.46131800	0.00021600
C	14.18663400	-1.57265000	0.00016700
C	12.80985200	-1.40749200	0.00015800
N	1.32760500	-1.74790900	-0.00025100
S	0.08159800	-2.84436100	-0.00037200
N	-1.21792800	-1.81323700	-0.00041600
O	-5.15275500	-1.86083600	-0.00034100
C	-5.64378900	-3.18228300	-0.00022700
O	-8.54900300	2.45724600	-0.00006300
C	-8.02873500	3.76859900	-0.00013100
O	8.02908800	-2.01214300	0.00004300
C	7.23210100	-3.17814700	-0.00001300
O	5.64474900	2.94857000	0.00012200
C	6.43290300	4.11736100	0.00018200
H	-1.29220300	2.78468200	-0.00024100
H	1.17892300	2.84543500	-0.00008100
H	-7.86969500	-1.97150400	-0.00008600
H	-5.83244000	2.55684100	-0.00032200
H	8.32004200	2.45817100	0.00018100
H	5.34963300	-1.51566400	-0.00002200
H	-12.21884600	-2.45600400	0.00038500
H	-14.68588900	-2.69543500	0.00066300
H	-16.12958700	-0.67790800	0.00078600
H	-15.10145100	1.58013900	0.00062900
H	-12.63148600	1.81891400	0.00035300
H	12.67929200	1.99581600	0.00028200
H	15.13886500	1.68929600	0.00029700
H	16.10588900	-0.59539800	0.00022300
H	14.60792900	-2.57343100	0.00013400
H	12.14564000	-2.26537700	0.00012000
H	-4.76205600	-3.82210600	-0.00026400
H	-6.24514600	-3.38443000	0.89487800
H	-6.24529300	-3.38453100	-0.89521100
H	-8.89216300	4.43390200	-0.00005900
H	-7.42292700	3.95899300	0.89418300
H	-7.42309700	3.95896700	-0.89456600
H	7.92854500	-4.01662500	-0.00001400
H	6.59891900	-3.22993600	0.89370900
H	6.59897400	-3.22988800	-0.89377700
H	5.73224500	4.95232500	0.00018900
H	7.06570300	4.17383600	0.89470900
H	7.06575300	4.17389500	-0.89430600

OPE-r’ (S0)

0 1

C	15.13872400	-0.09519100	0.00008000
C	14.44455500	1.11299100	0.00007500
C	13.05691600	1.12340300	0.00006300
C	12.34015800	-0.08513200	0.00005500
C	13.04813100	-1.29834200	0.00005900
C	14.43601900	-1.29820300	0.00007200
C	10.92109200	-0.07316200	0.00004300
C	9.70590700	-0.04091500	0.00003200
C	8.29231600	-0.02005900	0.00001900
C	7.59838400	1.21125800	0.00001400
C	6.21158500	1.21238800	0.00000100
C	5.48393600	0.01033000	-0.00000700
C	6.17802200	-1.22156500	-0.00000200
C	7.56623800	-1.22097200	0.00001100
C	4.07466800	0.02333100	-0.00002000
C	2.85620600	0.02508200	-0.00003300
C	1.45301800	0.01387900	-0.00004800
C	0.68528700	1.20309300	-0.00006700
C	-0.75659100	1.16465500	-0.00007000
C	-1.45713200	-0.06521100	-0.00006900
C	-0.69221800	-1.25941700	-0.00006500
C	0.75174300	-1.22079600	-0.00004400
C	1.32766500	2.47471900	-0.00006000
C	0.60610100	3.63308300	-0.00006900
C	-0.81370200	3.59342300	-0.00007600
C	-1.47224700	2.39781600	-0.00007800
N	-1.19121400	-2.50310500	-0.00006400
S	0.09001900	-3.49748900	-0.00004900
N	1.31478700	-2.43652500	-0.00003700
C	-15.13296300	0.25503700	0.00011200
C	-14.46191600	-0.96614300	0.00009800
C	-13.07474000	-1.00300900	0.00007700
C	-12.33511500	0.19168800	0.00006900
C	-13.01984400	1.41814900	0.00008300
C	-14.40749000	1.44445000	0.00010400
C	-10.91656000	0.15207500	0.00004700
C	-9.70229900	0.09438000	0.00002800
C	-8.28960900	0.04294100	0.00000600
C	-7.62201700	-1.20298300	-0.00000800
C	-6.23524100	-1.23422200	-0.00003000
C	-5.48419400	-0.04747300	-0.00003800
C	-6.15134000	1.19829400	-0.00002400
C	-7.53811000	1.22876100	-0.00000200
C	-4.07638500	-0.08847000	-0.00006200
C	-2.85884000	-0.10988700	-0.00007300
O	5.40969000	-2.32974600	-0.00001000
C	6.05705900	-3.57990600	-0.00000400
O	8.36706500	2.32454400	0.00002300
C	7.70925600	3.56912000	0.00002000
O	-5.35907300	2.29653100	-0.00003400
C	-5.98891500	3.55650000	-0.00001700
O	-8.41331300	-2.29897100	0.00000100
C	-7.78092000	-3.55790700	-0.00001200
H	16.22453000	-0.09918900	0.00008900
H	14.98886100	2.05268600	0.00008200
H	12.50751400	2.05906300	0.00006000
H	12.49498900	-2.23192200	0.00005300
H	14.97343600	-2.24184100	0.00007500
H	5.65382500	2.14038200	-0.00000100
H	8.12470200	-2.14849700	0.00001500
H	2.41267400	2.49220300	-0.00004500
H	1.11732000	4.59106500	-0.00006100
H	-1.37639700	4.52216100	-0.00008900
H	-2.55756300	2.35631900	-0.00008200
H	-16.21849300	0.27970300	0.00012800

H	-15.02398600	-1.89531200	0.00010400
H	-12.54322900	-1.94894000	0.00006600
H	-12.44909000	2.34107800	0.00007600
H	-14.92687000	2.39813900	0.00011400
H	-5.69299400	-2.17126100	-0.00004100
H	-8.07818200	2.16707800	0.00000900
H	5.26298000	-4.32673800	-0.00001200
H	6.67958600	-3.71234800	-0.89423900
H	6.67956800	-3.71234800	0.89424300
H	8.49455300	4.32562700	0.00002800
H	7.08541900	3.69695300	-0.89410500
H	7.08540400	3.69695000	0.89413400
H	-5.18645900	4.29478900	-0.00002500
H	-6.60903400	3.69733600	-0.89429300
H	-6.60900700	3.69732500	0.89428000
H	-8.58218100	-4.29739300	-0.00000200
H	-7.15987500	-3.69797900	-0.89388800
H	-7.15984700	-3.69798400	0.89384400

OPE-r’ (S1)

0 1

C	-15.07947500	-0.57049500	-0.00013500
C	-14.46374200	0.68020300	-0.00007400
C	-13.08047100	0.77895700	0.00000100
C	-12.28811000	-0.38357100	0.00001900
C	-12.91826000	-1.64095600	-0.00004700
C	-14.30252000	-1.72776300	-0.00012200
C	-10.87689400	-0.28220100	0.00010800
C	-9.66344100	-0.17396600	0.00018400
C	-8.26097900	-0.06302400	0.00015600
C	-7.64851800	1.21929500	0.00015900
C	-6.26689900	1.32189400	0.00013300
C	-5.45543900	0.17356100	0.00010500
C	-6.07082300	-1.11245800	0.00009500
C	-7.45311300	-1.21245300	0.00012300
C	-4.05900200	0.26015200	0.00007400
C	-2.83608800	0.27214800	0.00003300
C	-1.43970300	0.25808100	-0.00001000
C	-0.68189100	1.46918000	-0.00001500
C	0.75250900	1.42571700	-0.00001700
C	1.43270600	0.16884900	-0.00001500
C	0.67851100	-1.03494000	-0.00002000
C	-0.76086200	-0.99101500	-0.00001700
C	-1.31894600	2.73093300	-0.00001100
C	-0.59061000	3.89942300	-0.00001100
C	0.81638900	3.85506200	-0.00001100
C	1.47114300	2.64351300	-0.00001300
N	1.19250700	-2.26980500	-0.00002200
S	-0.10925600	-3.28805500	-0.00001600
N	-1.34679700	-2.19379300	-0.00001000
C	15.09400400	-0.19272900	0.00029400
C	14.35130300	-1.37242600	0.00030500
C	12.96521700	-1.32665500	0.00016900
C	12.29814400	-0.08804200	0.00002000
C	13.05585300	1.09674300	0.00000900
C	14.44168400	1.03906000	0.00014500
C	10.88383100	-0.04241500	-0.00011500
C	9.66575400	-0.02642600	-0.00021300
C	8.25908800	0.00571900	-0.00020100
C	7.51804100	-1.20586100	-0.00010000
C	6.13380000	-1.16314500	-0.00006600
C	5.44675300	0.06469800	-0.00013100
C	6.19011800	1.27765700	-0.00023500
C	7.57405800	1.23349600	-0.00026900
C	4.04885200	0.09904500	-0.00006000
C	2.82645000	0.11387300	0.00000100

O	-5.22318800	-2.15045600	0.00005500
C	-5.75679300	-3.45484900	0.00004700
O	-8.49461000	2.26884200	0.00018400
C	-7.93408300	3.56279600	0.00018500
O	5.45790300	2.41002100	-0.00028600
C	6.14677400	3.64012700	-0.00038300
O	8.24912500	-2.33796500	-0.00003500
C	7.55280300	-3.56560100	0.00007300
H	-16.16280500	-0.64317400	-0.00019300
H	-15.06715900	1.58295400	-0.00008500
H	-12.59108800	1.74729800	0.00005000
H	-12.30693200	-2.53741200	-0.00003500
H	-14.78002900	-2.70296100	-0.00017100
H	-5.77623800	2.28675500	0.00013300
H	-7.94306300	-2.17795000	0.00011600
H	-2.40422500	2.75495500	-0.00001100
H	-1.10346000	4.85658700	-0.00001000
H	1.38766700	4.77855100	-0.00000900
H	2.55643800	2.59915900	-0.00001000
H	16.17901700	-0.23325700	0.00040000
H	14.85733900	-2.33315800	0.00042000
H	12.37765800	-2.23876400	0.00017700
H	12.54123700	2.05203100	-0.00010700
H	15.01809900	1.95929100	0.00013500
H	5.53893700	-2.06754900	0.00002000
H	8.16455600	2.14087000	-0.00034100
H	-4.89664400	-4.12361600	0.00001500
H	-6.36449200	-3.63844400	0.89498900
H	-6.36453800	-3.63841500	-0.89486900
H	-8.77618100	4.25503000	0.00020500
H	-7.32257500	3.73516400	0.89453700
H	-7.32260500	3.73517800	-0.89418400
H	5.37924600	4.41413400	-0.00041100
H	6.77232500	3.74898000	0.89426900
H	6.77227600	3.74886600	-0.89508300
H	8.31648200	-4.34344700	0.00011200
H	6.92616800	-3.67106800	0.89402000
H	6.92612400	-3.67120000	-0.89382700

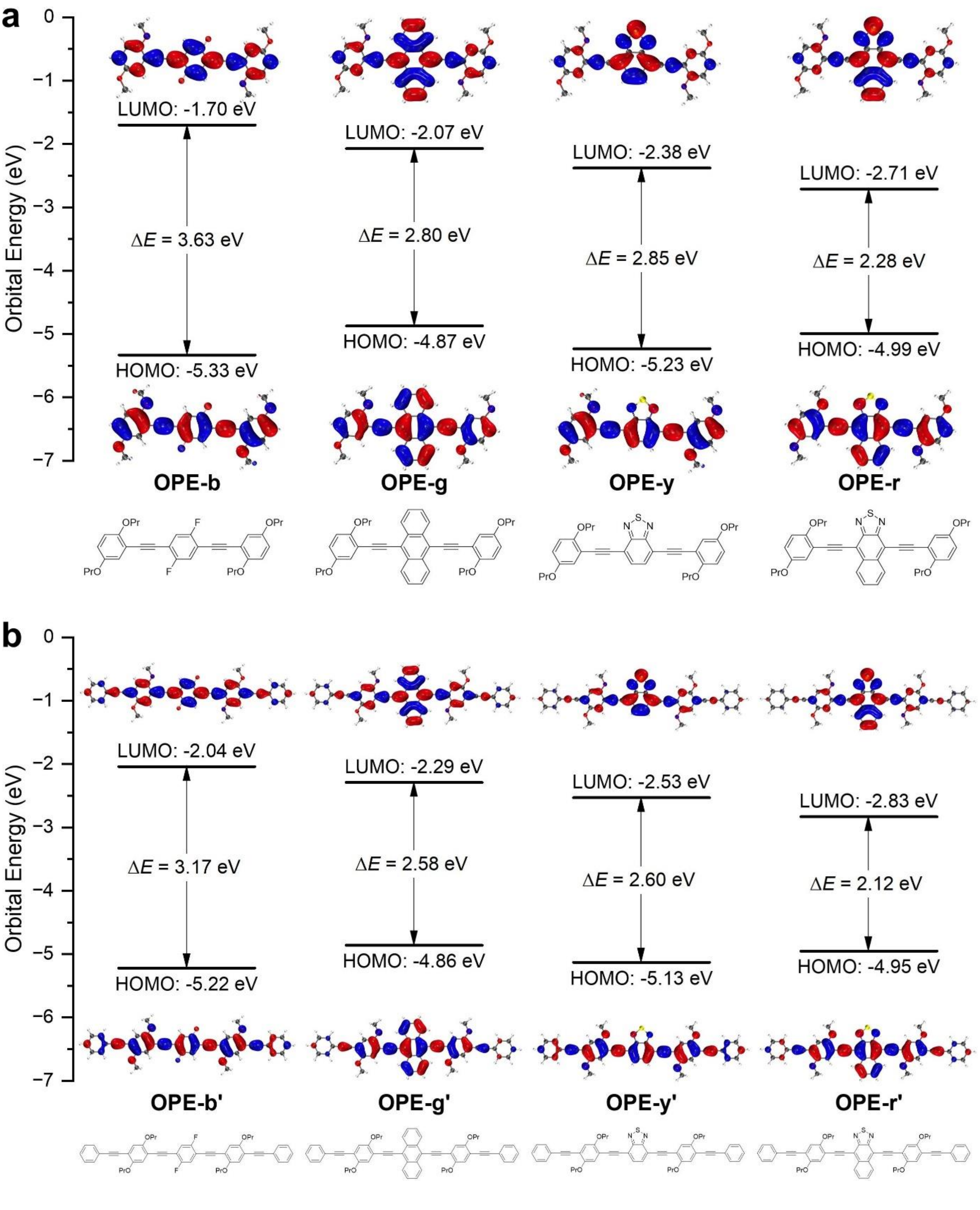

Figure S16. Calculated FMO energy levels and corresponding molecular orbital distributions for the optimized geometries of a) **OPE-*x*** und b) **OPE-*x*'** molecules. The calculations were performed at the D3-PBE0/6-31G** level of theory. HOMO: highest occupied molecular orbital; LUMO: lowest unoccupied molecular orbital. The orbital pictures are plotted with an isovalue of 0.02.

Table S1. Energy and oscillator strength of the calculated excited states.

Entry	State symbol	State energy / eV	Oscillator strength
**OPE-b**	T_1	2.30	0.00
	T_2	2.81	0.00
	T_3	3.23	0.00
	S_1	3.26	1.85
	T_4	3.56	0.00
	S_2	3.61	0.00
	T_5	3.78	0.00
	T_6	3.89	0.00
	T_7	3.92	0.00
	S_3	4.05	0.55
	T_8	4.13	0.00
	T_9	4.22	0.00
	T_{10}	4.26	0.00
	S_4	4.34	0.00
	S_5	4.46	0.09
	S_6	4.61	0.00
	S_7	4.64	0.00
	S_8	4.70	0.07
	S_9	4.74	0.00
	S_{10}	4.98	0.01
**OPE-g**	T_1	1.45	0.00
	S_1	2.63	1.42
	T_2	2.66	0.00
	T_3	2.72	0.00
	S_2	3.18	0.00
	T_4	3.27	0.00
	T_5	3.31	0.00
	S_3	3.32	0.02
	T_6	3.34	0.00
	T_7	3.39	0.00
	T_8	3.54	0.00
	T_9	3.60	0.00
	T_{10}	3.62	0.00
	S_4	3.71	0.00
	S_5	3.79	0.15
	S_6	3.88	0.00
	S_7	4.06	0.53
	S_8	4.18	0.00
	S_9	4.20	0.00
	S_{10}	4.21	0.00
**OPE-y**	T_1	1.63	0.00
	T_2	2.48	0.00
	S_1	2.49	0.91
	T_3	2.76	0.00
	S_2	2.88	0.00
	T_4	3.08	0.00
	T_5	3.22	0.00
	S_3	3.31	0.12
	T_6	3.37	0.00
	T_7	3.41	0.00
	T_8	3.61	0.00
	S_4	3.70	1.34
	T_9	3.79	0.00
	S_5	3.87	0.00
	T_{10}	3.87	0.00
	S_6	3.98	0.00
	S_7	3.99	0.00
	S_8	4.24	0.00
	S_9	4.29	0.04
	S_{10}	4.53	0.09

**OPE-r**	T_1	1.02	0.00
	S_1	2.10	0.90
	T_2	2.25	0.00
	T_3	2.39	0.00
	S_2	2.58	0.00
	T_4	2.81	0.00
	S_3	2.83	0.03
	T_5	2.99	0.00
	T_6	2.07	0.00
	T_7	3.12	0.00
	T_8	3.24	0.00
	S_4	3.41	0.69
	T_9	3.41	0.00
	T_{10}	3.50	0.00
	S_5	3.52	0.01
	S_6	3.58	0.68
	S_7	3.66	0.00
	S_8	3.75	0.00
	S_9	3.89	0.06
	S_{10}	4.10	0.06
**OPE-b'**	T_1	2.02	0.00
	T_2	2.30	0.00
	S_1	2.78	3.67
	T_3	2.85	0.00
	T_4	3.12	0.00
	T_5	3.18	0.00
	S_2	3.21	0.00
	T_6	3.32	0.00
	T_7	3.49	0.00
	S_3	3.59	0.00
	T_8	3.63	0.00
	T_9	3.71	0.00
	S_4	3.79	0.77
	S_5	3.85	0.11
	T_{10}	3.86	0.00
	S_6	3.95	0.00
	S_7	4.01	0.08
	S_8	4.24	0.09
	S_9	4.42	0.02
	S_{10}	4.46	0.00
**OPE-g'**	T_1	1.38	0.00
	T_2	2.22	0.00
	S_1	2.33	2.96
	T_3	2.34	0.00
	T_4	2.84	0.00
	S_2	2.86	0.00
	T_5	2.99	0.00
	S_3	3.08	0.02
	T_6	3.14	0.00
	T_7	3.19	0.00
	T_8	3.24	0.00
	S_4	3.25	0.00
	T_9	3.30	0.00
	T_{10}	3.30	0.00
	S_5	3.41	0.26
	S_6	3.67	0.01
	S_7	3.68	0.24
	S_8	3.70	0.00
	S_9	3.88	0.95
	S_{10}	4.03	0.00

**OPE-y'**	T_1	1.53	0.00
	T_2	2.15	0.00
	S_1	2.26	2.11
	T_3	2.43	0.00
	S_2	2.67	0.00
	T_4	2.75	0.00
	T_5	2.87	0.00
	T_6	3.09	0.00
	T_7	3.13	0.00
	S_3	3.21	1.99
	T_8	3.26	0.00
	S_4	3.28	0.02
	T_9	3.30	0.00
	T_{10}	3.38	0.00
	S_5	3.40	0.00
	S_6	3.54	0.02
	S_7	3.55	0.00
	S_8	3.76	0.00
	S_9	3.90	0.33
	S_{10}	3.96	0.00
**OPE-r'**	T_1	0.97	0.00
	S_1	1.92	1.90
	T_2	1.99	0.00
	T_3	2.14	0.00
	S_2	2.38	0.00
	T_4	2.55	0.00
	T_5	2.58	0.00
	S_3	2.76	0.18
	T_6	2.90	0.00
	T_7	2.94	0.00
	S_4	2.99	1.50
	T_8	3.00	0.00
	T_9	3.00	0.00
	S_5	3.12	0.05
	T_{10}	3.18	0.00
	S_6	3.19	0.30
	S_7	3.33	0.00
	S_8	3.65	0.18
	S_9	3.65	0.00
	S_{10}	3.73	0.02

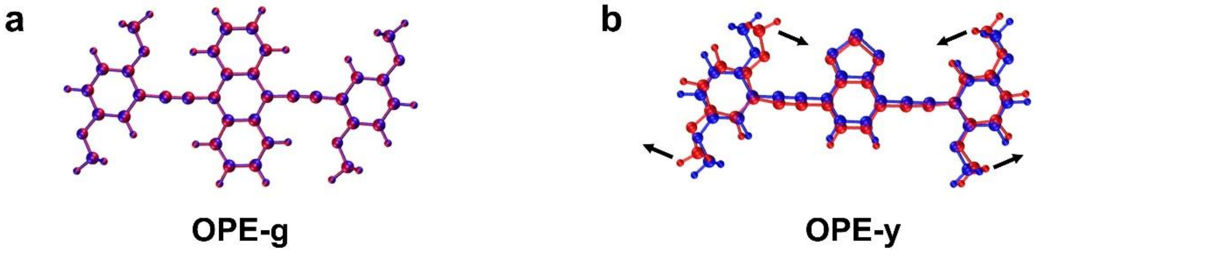

Figure S17. Overlayed structures of optimized S_0 (blue) geometry and S_1 (red) geometry for a) **OPE-g** and b) **OPE-y**.

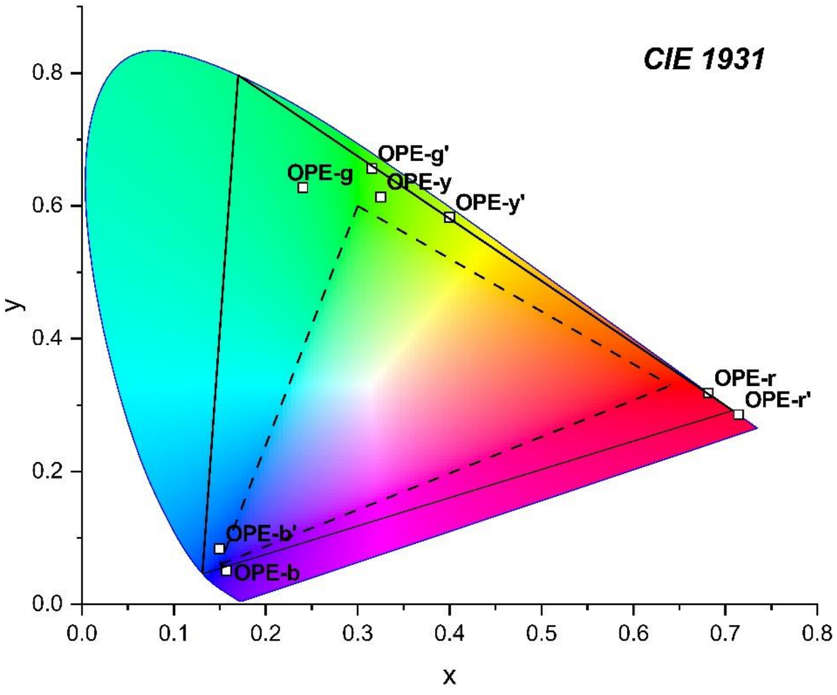

Figure S18. Emission color coordinates of **OPE-*x*** and **OPE-*x’*** plotted on the CIE 1931 diagram. The dashed triangle denotes the standard sRGB color gamut,[10] while the solid black triangle outlines the wider BT.2020 color gamut.[11]

6 References

1. Lee, O. S. & Zysman-Colman, E. Digichem (Version 7). Chemicus Limited (2025).

2. Lee, O. S., Gather, M. C. & Zysman-Colman, E. Digichem: computational chemistry for everyone. *Digital Discovery* **3**, 1695–1713 (2024).

3. O’boyle, N. M., Tenderholt, A. L. & Langner, K. M. cclib: A library for package-independent computational chemistry algorithms. *J. Comput. Chem.* **29**, 839–845 (2008).

4. Humphrey, W., Dalke, A. & Schulten, K. VMD: Visual molecular dynamics. *J. Mol. Graph.* **14**, 33–38 (1996).

5. Stone, J. E. An efficient library for parallel ray tracing and animation. Master's thesis (University of Missouri-Rolla, Rolla, MO, 1998).

6. Hunter, J. D. Matplotlib: A 2D Graphics Environment. *Comput. Sci. Eng.* **9**, 90–95 (2007).

7. O’Boyle, N. M. *et al.* Open Babel: An open chemical toolbox. *J. Cheminform.* **3**, 33 (2011).

8. O’Boyle, N. M., Morley, C. & Hutchison, G. R. Pybel: a Python wrapper for the OpenBabel cheminformatics toolkit. *Chem. Cent. J.* **2**, 5 (2008).

9. Gao, X. *et al.* Evaluation of Spin-Orbit Couplings with Linear-Response Time-Dependent Density Functional Methods. *J. Chem. Theory Comput.* **13**, 515–524 (2017).

10. Stokes, M., Anderson, M., Chandrasekar, S. & Motta, R. A Standard Default Color Space for the Internet - sRGB. https://www.w3.org/Graphics/Color/sRGB.html (1996). Accessed 6 June 2026.

11. BT.2020 : Parameter values for ultra-high definition television systems for production and international programme exchange. *ITU-R.* https://www.itu.int/rec/R-REC-BT.2020-2-201510-I/en (2015). Accessed 6 June 2026.
